# Piezo1 agonist restores meningeal lymphatic vessels, drainage, and brain-CSF perfusion in craniosynostosis and aged mice

**DOI:** 10.1172/JCI171468

**Published:** 2023-11-02

**Authors:** Matt J. Matrongolo, Phillip S. Ang, Junbing Wu, Aditya Jain, Joshua K. Thackray, Akash Reddy, Chi Chang Sung, Gaëtan Barbet, Young-Kwon Hong, Max A. Tischfield

**Affiliations:** 1 Department of Cell Biology and Neuroscience, Rutgers University, Piscataway, New Jersey, USA.; 2 Child Health Institute of New Jersey, Robert Wood Johnson Medical School, New Brunswick, New Jersey, USA.; 3 Department of Genetics and the Human Genetics Institute of New Jersey, Rutgers, The State University of New Jersey, Piscataway, New Jersey, USA.; 4 Department of Pediatrics and; 5 Department of Pharmacology, Robert Wood Johnson Medical School, New Brunswick, New Jersey, USA.; 6 Department of Surgery, Norris Comprehensive Cancer Center, Keck School of Medicine, University of Southern California, Los Angeles, California, USA.; 7 Department of Biochemistry and Molecular Medicine, Norris Comprehensive Cancer Center, Keck School of Medicine, University of Southern California, Los Angeles, California, USA

**Keywords:** Neuroscience, Vascular Biology, Genetic diseases, Mouse models

## Abstract

Skull development coincides with the onset of cerebrospinal fluid (CSF) circulation, brain-CSF perfusion, and meningeal lymphangiogenesis, processes essential for brain waste clearance. How these processes are affected by craniofacial disorders such as craniosynostosis are poorly understood. We report that raised intracranial pressure and diminished CSF flow in craniosynostosis mouse models associate with pathological changes to meningeal lymphatic vessels that affect their sprouting, expansion, and long-term maintenance. We also show that craniosynostosis affects CSF circulatory pathways and perfusion into the brain. Further, craniosynostosis exacerbates amyloid pathology and plaque buildup in *Twist1^+/–^:5xFAD* transgenic Alzheimer’s disease models. Treating craniosynostosis mice with Yoda1, a small molecule agonist for Piezo1, reduces intracranial pressure and improves CSF flow, in addition to restoring meningeal lymphangiogenesis, drainage to the deep cervical lymph nodes, and brain-CSF perfusion. Leveraging these findings, we show that Yoda1 treatments in aged mice with reduced CSF flow and turnover improve lymphatic networks, drainage, and brain-CSF perfusion. Our results suggest that CSF provides mechanical force to facilitate meningeal lymphatic growth and maintenance. Additionally, applying Yoda1 agonist in conditions with raised intracranial pressure and/or diminished CSF flow, as seen in craniosynostosis or with ageing, is a possible therapeutic option to help restore meningeal lymphatic networks and brain-CSF perfusion.

## Abstract 2

<p>The Piezo1 agonist Yoda1 acts to restore meningeal lymphatic vessels, lymph node drainage, and brain-CSF perfusion in craniosynostosis and aged mice.</p>

## Graphical abstract

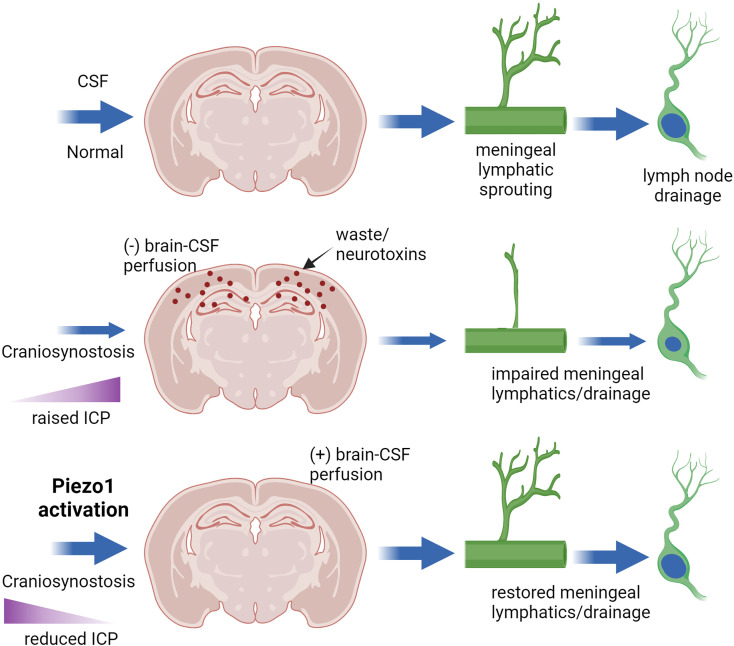

## Introduction

Meningeal lymphatic vessels (MLVs) facilitate drainage of cerebrospinal fluid (CSF) from the head to help control CNS waste clearance, immune surveillance, and responses to injury (1). MLVs reside in dura, where they grow along the venous sinuses in close apposition to the skull. They express transcription factors and cell surface markers common to lymphatic vessels. However, the growth of MLVs in mice occurs exclusively during the first postnatal weeks, and in a basal to dorsal progression from the base to the top of the skull, mirroring establishment of CSF circulation routes (2–4). VEGF-C/VEGFR3 signaling is required for meningeal lymphangiogenesis, but, unlike most peripheral lymphatic vessels, MLVs require continuous VEGF-C signaling to maintain vessel integrity and survival (2). MLVs are further distinguished from peripheral lymphatics according to transcriptional regulation (5). These data suggest the growth and maintenance of MLVs are shaped by processes and environmental factors unique to the meningeal environment.

Growth and expansion of lymphatic networks is dependent upon interstitial fluid and laminar flow (6–8). Laminar flow helps maintain the integrity of mature lymphatic networks via activation of Piezo1 mechanotransduction signaling (7). MLVs that grow alongside the venous sinuses have access to CSF and, thus, exposure to laminar flow (9–11). Injury models that impede CSF drainage to the deep cervical lymph nodes (dCLNs) are associated with pathological changes to MLVs, potentially because these vessels try to compensate for loss of flow (12, 13). As CSF flow and turnover naturally declines with age (14, 15), dorsal MLVs along the superior sagittal sinus (SSS) and confluence regress, whereas vessels found at the skull base become hyperplastic and take on a lymphedematous-like appearance (16, 17). These observations suggest that CSF may help guide meningeal lymphangiogenesis and long-term maintenance to facilitate CNS waste clearance and immune surveillance.

Restoring the functional integrity of MLVs in pathological conditions or with ageing has important clinical implications. In aged animals, rejuvenation of MLVs with adenoviral delivery of VEGF-C improves brain-CSF perfusion, drainage of macromolecules from CSF, and normal cognitive functions (17). In traumatic brain injury models, this application also limits the extent of Iba1 gliosis (12). In Alzheimer’s disease (AD) models, augmenting MLV functions with VEGF-C enhances the effectiveness of monoclonal antibodies that target amyloid-β to clear plaques from the brain (18). In intracranial tumor models, improving MLV drainage with VEGF-C promotes survival and generates immunogenic responses against tumors (19, 20). Thus, enhancing MLV functions in ageing and/or disease states improves waste clearance and immune surveillance, underscoring the need to characterize factors that control the growth, maturation, and maintenance of these vessels.

We previously investigated MLV growth and expansion in *Twist1^fl/fl^:Sm22a-Cre* mouse models for craniosynostosis (CS), a craniofacial disorder that affects skull growth via premature fusion of cranial sutures (21, 22). In humans, loss-of-function mutations in *TWIST1* or gain-of-function mutations in *FGFR2* cause syndromic forms of CS that are associated with dural venous sinus malformations and raised intracranial pressure (ICP) (23–25). We showed the growth and sprouting of dorsal MLVs along the transverse sinuses (TVS) was reduced in *Twist1^fl/fl^:Sm22a-Cre* mice, whereas basal vessels were hyperplastic in some animals. Drainage to the dCLNs was also diminished (22). Insults to the dural extracellular matrix and/or loss of growth factor signaling from venous smooth muscle was postulated to affect MLV development.

We now show that pathological changes to MLVs in CS can occur without affection to dura or the venous sinuses. Instead, these are associated with raised ICP and diminished CSF flow to perisinusoidal dura. In addition to reduced MLV sprouting and expansion, adult CS mice show premature regression of dorsal MLVs and hyperplastic basal vessels, suggesting that MLVs are exposed to conditions that induce precocious ageing. Reduced drainage of CSF macromolecules to the dCLNs associates with reduced brain-CSF perfusion. Additionally, inducing CS in 5xFAD models for familial AD increases amyloid-β buildup. Treating CS model mice (CS mice) with Yoda1, a selective Piezo1 agonist, reduces ICP and helps restore MLV functions and brain-CSF perfusion. Finally, Yoda1 treatment in aged mice can also improve MLV coverage, drainage, and brain-CSF perfusion. Our results suggest raised ICP in CS restricts MLV access to CSF and affects the development, maintenance, and functional drainage of MLVs, while also hindering brain-CSF perfusion and macromolecule clearance. Thus, MLVs appear sensitive to changes in laminar flow, suggesting that CSF may provide mechanical force to facilitate the development, integration, and maintenance of CNS waste clearance systems across the lifespan.

## Results

### ICP is increased in Twist1 CS mouse models.

**Figure 1:**
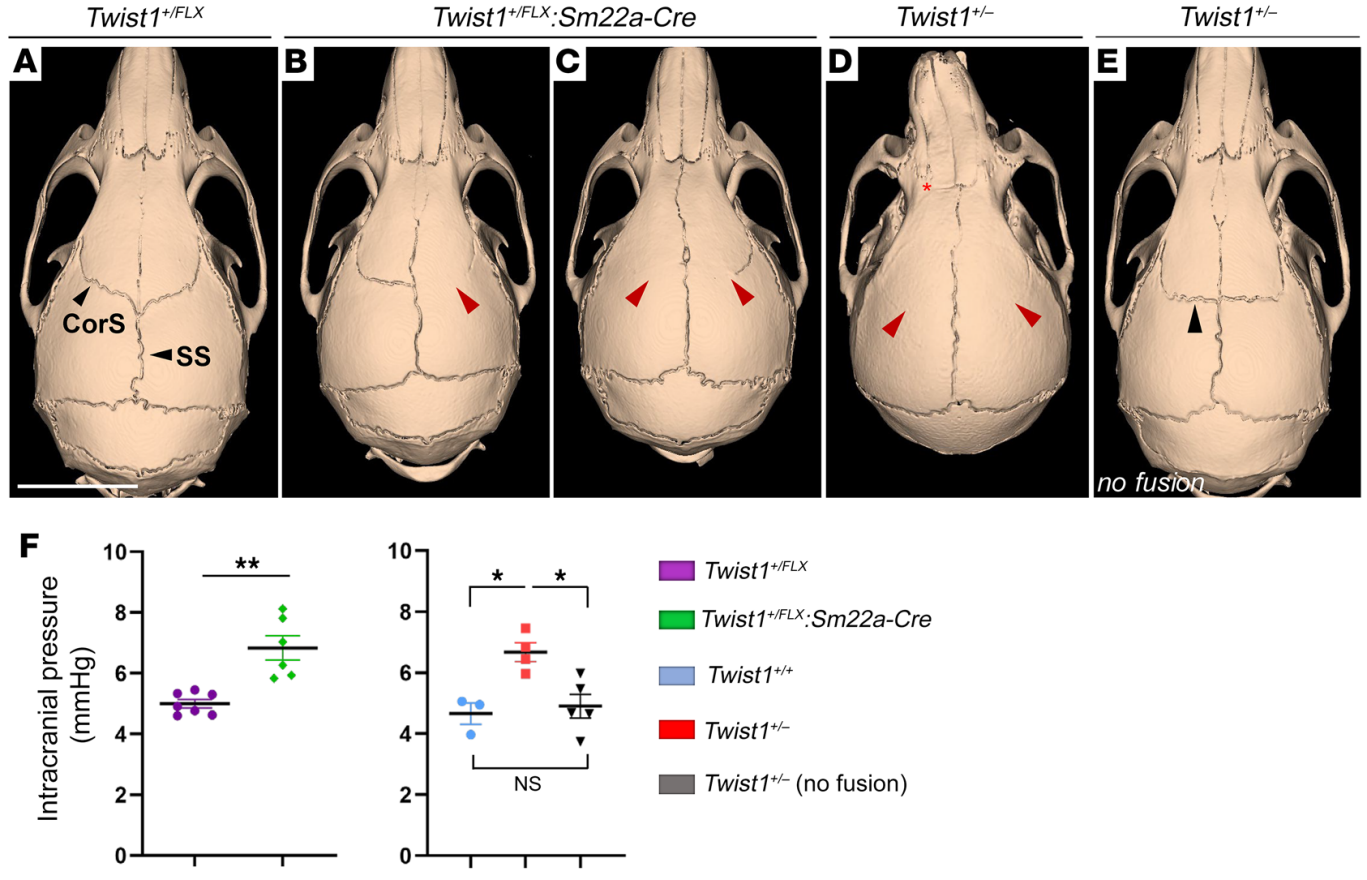
Twist1 haploinsufficiency in cranial sutural mesenchyme causes CS and raised ICP. Representative reconstructed CT scans from 2-month-old adults showing normal skull and suture morphology in a *Twist1^+/FLX^* control (**A**) versus *Twist1^+/FLX^:Sm22a-Cre* (**B** and **C**) and *Twist1^+/–^* mice with (**D**) or without (**E**) suture fusion. (**B**) Near-complete unilateral fusion of the right coronal suture (red arrowhead). (**C**) Partial bilateral fusion with full and partial fusion on the left and right coronal sutures, respectively (red arrowheads). (**D**) Complete bilateral fusion (red arrowheads) accompanied by fusion of the frontonasal suture (asterisk) and a deviated nasal bone. (**E**) *Twist1^+/–^* mouse without suture fusion. The orientation of the coronal sutures is flatter and more ‘box-like’ (black arrowhead). (**F**) ICP is raised in *Twist1^+/FLX^:Sm22a-Cre* and *Twist1^+/–^* mice with CS but is normal in *Twist1^+/–^* mice without suture fusion [*Twist1^+/FLX^* (*n* = 7); *Twist1^+/FLX^:Sm22a-Cre* (*n* = 6); *Twist1^+/+^* (*n* = 3); *Twist1^+/–^* (*n* = 4); *Twist1^+/–^* no fusion (*n* = 5)]. CorS, coronal suture; SS, sagittal suture. *P ≤ 0.05, **P ≤ 0.01. 2-tailed unpaired *t* test with Welch’s correction (left) 1-way ANOVA with Tukey’s multiple comparison test (right). Scale bar: 5 mm.

Raised ICP is a primary concern in CS, causing neurological complications and cognitive deficits (24, 25). Injury models show that raised ICP is associated with transient, pathological changes to MLVs that hinder drainage (12, 13). However, the effects of pathological, chronically raised ICP — as seen in conditions such as CS — on MLV development and drainage have not been investigated. We addressed this by inactivating a single copy of *Twist1* using *Sm22a-Cre*, both of which are expressed in periosteal dura and sutural mesenchyme, but not MLVs (22, 23). Approximately 56% (18 of 32) of *Twist1^+/FLX^:Sm22a-Cre* adult mice had unilateral or bilateral coronal synostosis, with partial or full fusion of the sutures, the former more common (Figure 1, A–C). ICP was significantly increased in young adults aged 2–4 months, similar to previous findings in *Twist1^+/–^* mice, which are also used to model syndromic CS (26) (Figure 1F). In agreement, ICP was also increased in our *Twist1^+/–^* mouse models aged 2–4 months (Figure 1F). Coronal synostosis was more penetrant in *Twist1^+/–^* mice (approximately 76%, or 23 of 30), with a greater proportion showing full unilateral and/or bilateral synostoses (Figure 1D). In *Twist1^+/–^* mice without suture fusion, ICP was normal (Figure 1, E and F). Thus, both *Twist1^+/FLX^:Sm22a-Cre* and *Twist1^+/–^* mice can have CS associated with raised ICP.

### CS affects MLV growth and sprouting in the absence of dura and venous sinus malformations.

We previously reported that sprouting and expansion of MLVs along the TVS was reduced in homozygous *Twist1^fl/fl^:Sm22a-Cre* mice. These mice are more severely affected than *Twist1^+/FLX^:Sm22a-Cre* and *Twist1^+/–^* mice, with regionalized absence of mineralized bone in the calvarium, hypoplastic dura, and loss or hypoplasia of the TVS (22). We speculated that hypoplasia of the dural extracellular matrix and/or loss of growth factor signaling from venous smooth muscle may have exerted deleterious effects on MLVs.

The TVS were not missing nor hypoplastic in *Twist1^+/FLX^:Sm22a-Cre* and *Twist1^+/–^* mice and showed normal smooth muscle coverage, the latter of which is also consistent with *Twist1^fl/fl^:Sm22a-Cre* homozygotes (Supplemental Figure 1, A–C; supplemental material available online with this article; https://doi.org/10.1172/JCI171468DS1) (22). The dura and arachnoid membranes, as labeled by Crabp2, appeared qualitatively normal at the dorsal midline in *Twist1^+/–^* newborns (Supplemental Figure 1D). In juveniles and adults, the dural membrane was not hypoplastic and could be peeled off the skull in 1 piece (Supplemental Figure 1E), which was impossible in *Twist1^fl/fl^:Sm22a-Cre* homozygotes due to its hypoplastic state. Given the absence of overt changes to dura and surrounding venous vasculature seen in *Twist1^fl/fl^:Sm22a-Cre* mice, we asked whether MLV growth and sprouting was also affected in *Twist1^+/FLX^:Sm22a-Cre* and *Twist1^+/–^* mice.

**Figure 2:**
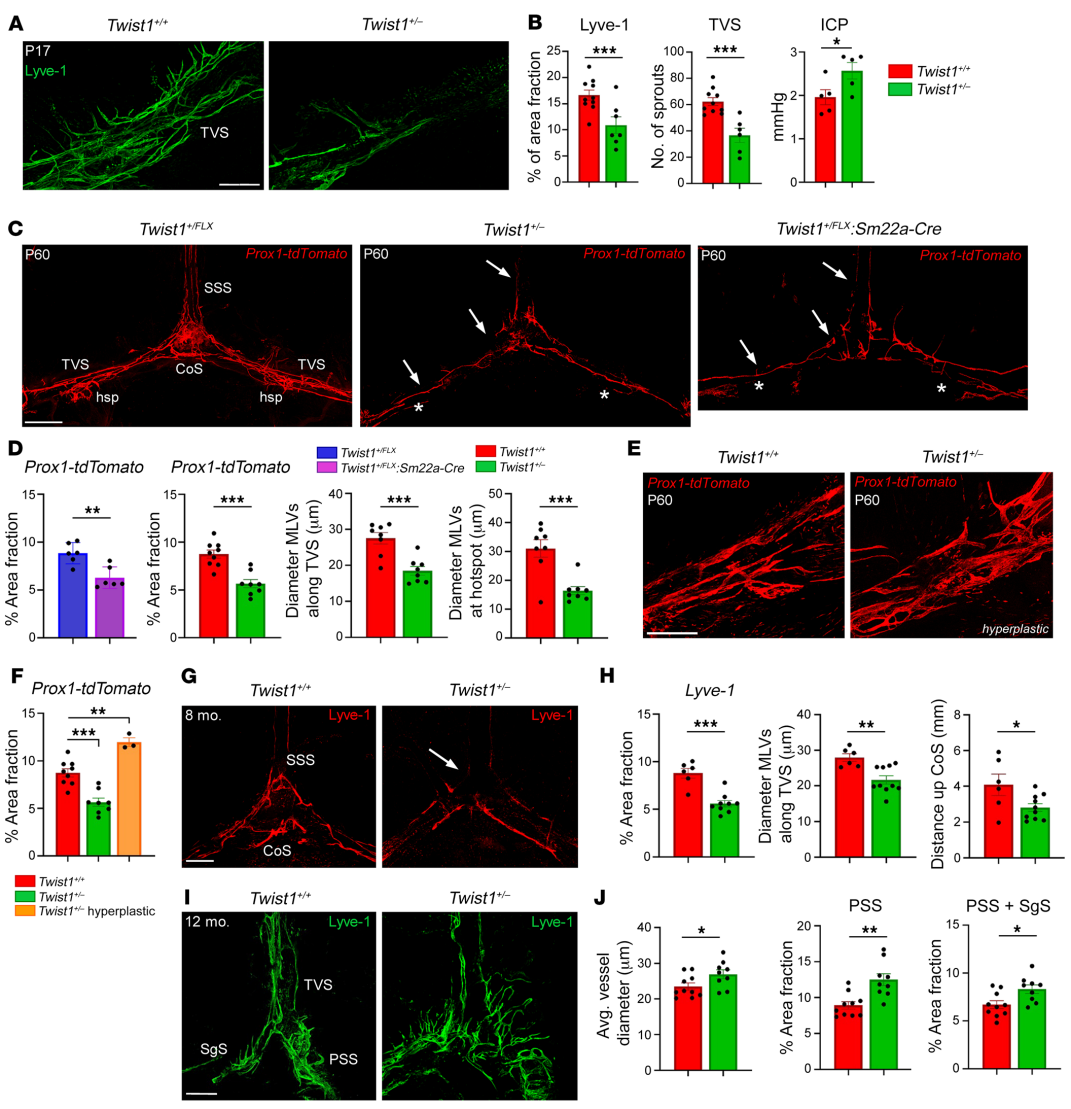
CS affects MLV sprouting, expansion, and long-term maintenance. (**A**) Representative images from P17 mice. Sprouting and expansion of MLVs along the TVS is reduced in mice with CS and raised ICP. (**B**) Quantification of percent area fraction of Lyve-1 [*Twist1^+/+^* (*n* = 10); *Twist1^+/–^* (*n* = 7)], number of sprouts [*Twist1^+/+^* (*n* = 10); *Twist1^+/–^* (*n* = 6)], and levels of ICP at P17 [*n* = 5/genotype]. (**C**) Compared with controls (left), MLVs along the SSS and TVS (arrows) are hypoplastic in *Twist1^+/FLX^:Sm22a-Cre:Prox1-tdTomato* (right) and *Twist1^+/–^:Prox1-tdTomato* mice (center). Hotspots along the TVS are poorly formed or missing (asterisks). (**D**) Quantification of percent area fraction [*Twist1^FLX/+^* and *Twist1^FLX/+^:Sm22a-Cre* (*n* = 6); *Twist1^+/+^* (*n* = 9) and *Twist1^+/–^* (*n* = 8)] and average vessel diameters along the TVS [*Twist1^+/+^* and *Twist1^+/–^* (*n* = 8)] and at hotspots [*Twist1^+/+^* and *Twist1^+/–^* (*n* = 8)]. (**E** and **F**) A small subset of CS mice shows signs of vessel hyperplasia [*Twist1^+/–^* (*n* = 3)]. (**G**) In 8–10-month-old adults, dorsal MLVs are further regressed, especially at the sinus confluence and along the SSS (arrow). (**H**) Quantification of Lyve-1 percent area fraction [*Twist1^+/+^* (*n* = 6); *Twist1^+/–^* (*n* = 9)], average vessel diameter along the TVS [*Twist1^+/+^* (*n* = 6); *Twist1^+/–^* (*n* = 10)], and growth from the CoS [*Twist1^+/+^* (*n* = 6); *Twist1^+/–^* (*n* = 10)]. (**I**) Basal MLVs along the SgS and PSS are hyperplastic in *Twist1^+/–^* mice with CS. (**J**) Quantification of average vessel diameter and the percent area fraction of MLVs along the PSS and SgS [*Twist1^+/+^* (*n* = 10); *Twist1^+/–^* (*n* = 9)]. SSS, superior sagittal sinus; TVS, transverse sinus; CoS, confluence of sinuses; hsp, hotspot; SgS, sigmoid sinus; PSS, petrosquamosal sinus. *P ≤ 0.05, **P ≤ 0.01, ***P ≤ 0.001 2-tailed unpaired *t* test (**B**, **D**, **H**, and **J**), 1-way ANOVA with Tukey’s multiple comparison test (**F**). Scale bars: 200 μm (**A**); 1 mm (**C**); and 500 μm (**E**, **G**, and **I**).

Numerous loops and sprouts were present in control mice along the TVS at postnatal day 17 (P17), especially at or near regions where ‘hotspots’ are found in adults (Figure 2A). Hotspots are important for immune cell and macromolecule uptake and show increased branching and complexity along the TVS and at the confluence (5). Interestingly, MLV networks were affected in both *Twist1^+/FLX^:Sm22a-Cre* and *Twist1^+/–^* mice, similar to homozygous *Twist1^fl/fl^:Sm22a-Cre* mice. MLV coverage and average vessel diameter was reduced along the TVS, as was growth at the sinus confluence and SSS. Sprouting along the TVS was significantly reduced, especially at locations where hotspots were forming (Figure 2, A and B). Raised ICP was also evident in mice with CS by this time (Figure 2B). Thus, CS perturbs the growth and sprouting of MLVs, even in the absence of dura and venous sinus malformations.

### MLVs show morphological changes in young adult mice with CS and raised ICP.

Previous findings in 2–3-month-old *Twist1^fl/fl^:Sm22a-Cre* mice revealed hypoplastic MLV networks along the TVS and SSS (22). In 2–4-month-old *Twist1^+/FLX^:Sm22a-Cre* and *Twist1^+/–^* mice with bilateral, full unilateral, or partial unilateral (at least 50%) coronal synostosis, dorsal networks along the TVS and SSS were also hypoplastic with reduced branching and complexity (Figure 2, C and D). Hotspots along the TVS were either hypoplastic or missing on one or both sides (Figure 2C). Vessel complexity and area coverage at the sinus confluence and along the SSS were also reduced. By contrast, MLVs were normal in mutant mice without synostosis and raised ICP (Supplemental Figure 2, A and C). Interestingly, MLVs along the pterygopalatine and middle meningeal arteries appeared normal despite poor development of neighboring vessels patterned along the venous sinuses (Supplemental Figure 1, F and G). These results suggest MLVs along arterial vessels are spared in CS, and that their development and/or maintenance might be differentially regulated from those that grow along the venous sinuses.

Next, we asked if MLVs show pathological changes in different genetic forms of CS. We developed a model for Apert Syndrome using mice that permit conditional activation of a constitutively active Fgfr2 receptor (*Fgfr2^S252W^*), the most common mutation found in syndromic CS (27). *Fgfr2^+/S252W^:Sm22a-Cre* adult mice had full to partial fusion of the sagittal suture and full to partial — unilateral or bilateral — fusion of the coronal sutures (Supplemental Figure 3A). ICP was significantly increased in adults (Supplemental Figure 3B). Furthermore, dorsal MLV networks were hypoplastic and also missing hotspots (Supplemental Figure 3, C and D). Thus, pathological changes to MLVs occur in other forms of CS with raised ICP.

### Dorsal MLV networks prematurely regress as CS mice age.

The majority of 2–4-month-old adult mice with full unilateral or partial/full bilateral fusion had hypoplastic dorsal networks. On rare occasions, some mice showed signs of vessel hyperplasia at the sinus confluence and/or at hotspots along the TVS (Figure 2, E and F). Interestingly, following blunt trauma, acutely raised ICP causes transient increases in vessel coverage and the numbers of loops and sprouts around hotspots (12). This suggests that dorsal MLV networks are undergoing pathological changes in CS that may be influenced by environmental stressors (i.e., ICP). Considering that these pathological changes may continue to progress, we characterized the effects of chronic ICP on dorsal MLVs in middle-aged *Twist1^+/–^* mice at 8–10 months. Compared with younger adults, these networks were more affected. Hotspots along the TVS were rudimentary or completely missing and vessel coverage at the sinus confluence and proximally along the SSS was further reduced (Figure 2, G and H). Remarkably, these phenotypes are similar to mice aged approximately 20–24 months (16, 17), although they are present at least 1 year earlier in CS models. Thus, dorsal MLVs continue to regress and age prematurely in CS.

### Basal MLVs demonstrate early onset hyperplasia in CS.

MLVs near the skull base along the petrosquamousal and sigmoid sinuses are a major outflow route for CSF (Supplemental Figure 1H) (16). Although dorsal networks show regression in aged mice, basal lymphatics become hyperplastic for unknown reasons. We previously reported that basal networks were hyperplastic in some *Twist1^fl/fl^:Sm22a-Cre* mice (22). In 2–4-month-old adult CS mice, basal networks appeared relatively normal, despite affection to dorsal networks. However, basal networks were hyperplastic in middle-aged *Twist1^+/–^* mice (8–12 months), especially along the petrosquamosal sinus (Figure 2, I and J). This result is striking because WT mice only show this phenotype by approximately 24 months of age (16), at which time CSF flow/turnover is reduced and drainage to the dCLNs declines (14, 17). Thus, MLVs in middle-aged mice with CS show signs of precocious ageing, with premature regression of dorsal vessels coupled with hyperplastic basal networks.

### CSF flow to perisinusoidal dura and the dCLNs is reduced in CS.

**Figure 3:**
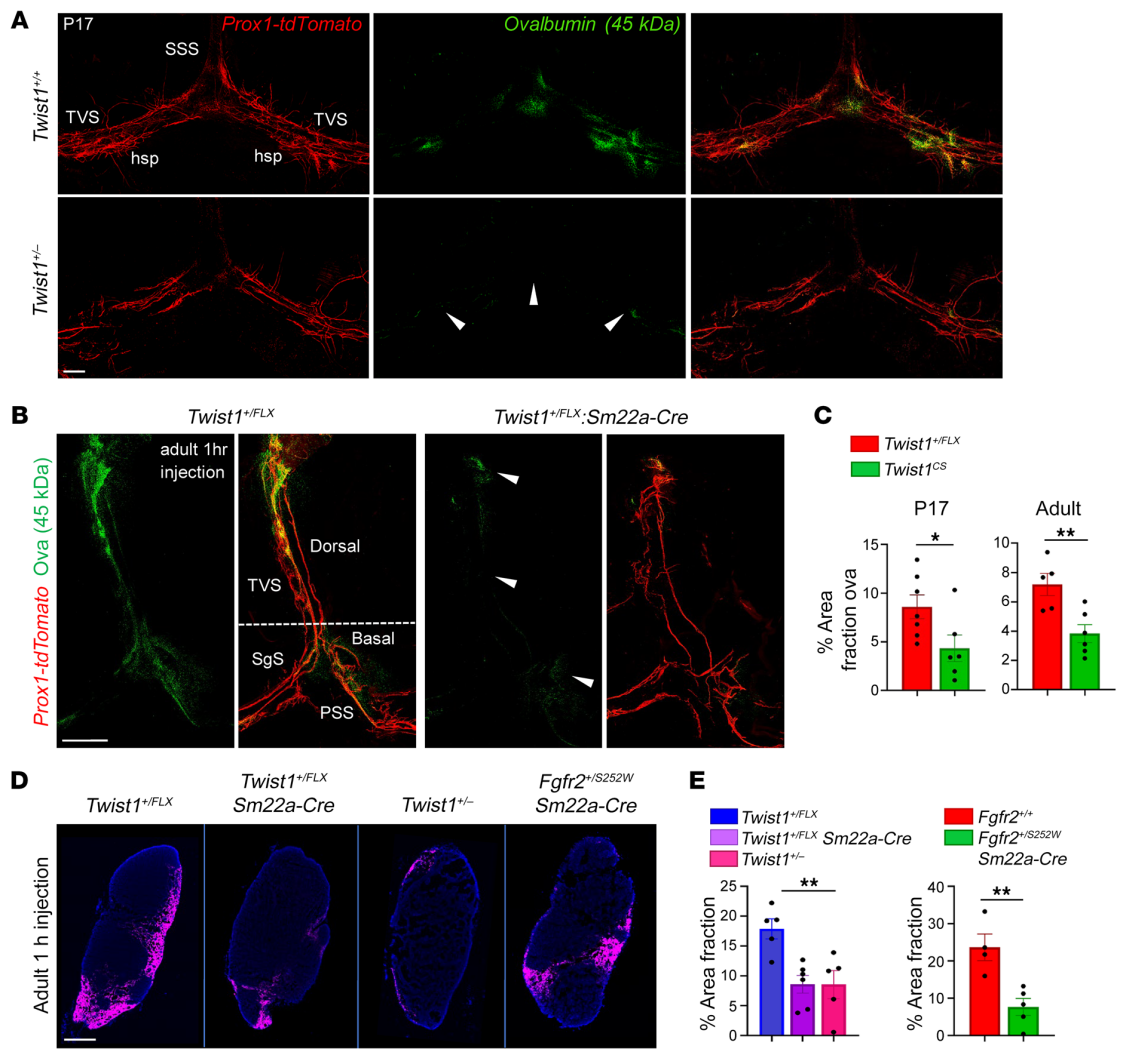
CSF flow to perisinusoidal dura and the dCLNs is reduced in mice with CS. (**A** and **C**) Following injection of 45 kDa ovalbumin tracer into the cisterna magna, juvenile P17 mice with *Twist1* haploinsufficiency and CS (bottom panel in **A**) show a significant reduction in tracer deposition in perisinusoidal dura compared to unaffected littermates (top panel), especially at regions where hotspots form (arrowheads) [*Twist1^+/+^* (*n* = 7); *Twist1^+/–^* (*n* = 6)]. (**B** and **C**) In 2–4-month-old adults, tracer deposition in perisinusoidal dura surrounding dorsal MLVs is significantly reduced. Flow to perisinusoidal dura surrounding basal vessels is also reduced (arrowheads) [*Twist1^FLX/+^* (*n* = 5); *Twist1^FLX/+^:Sm22a-Cre* (*n* = 6)]. See Supplemental Figure 1H for additional orientation. (**D**) Representative images show that tracer drainage to the dCLNs is significantly reduced in *Twist1^+/FLX^:Sm22a-Cre*, *Twist1^+/–^*, and *Fgfr2^+/S252W^:Sm22a-Cre* mice with CS. Pink shows the 45kDa ovalbumin tracer and blue is Dapi. (**E**) Quantification of percent area fraction of ovalbumin tracer in dCLNs [*Twist1^FLX/+^* (*n* = 5); *Twist1^FLX/+^:Sm22a-Cre* (*n* = 6); *Twist1^+/–^* (*n* = 5); *Fgfr2^+/+^* (*n* = 4); *Fgfr2^+/S252W^:Sm22a-Cre* (*n* = 5)]. SSS, superior sagittal sinus; TVS, transverse sinus; hsp, hotspot; SgS, sigmoid sinus; PSS, petrosquamosal sinus. *P ≤ 0.05, **P ≤ 0.01 2-tailed unpaired *t* test (**C**; **E**, right). 1-way ANOVA with Tukey’s multiple comparison test (**E**, left). Scale bars: 500 μm (**A**); 200 μm (**B**); 200 μm (**D**).

Growth and sprouting of peripheral lymphatics require increasing ISF and laminar flow (7, 8). We hypothesized CS and raised ICP may affect the flow of CSF to perisinusoidal dura, impinging upon the development of MLVs along the venous sinuses, which receive access to CSF. We injected a 45 kDa ovalbumin tracer into the cisterna magna of *Twist1^+/FLX^:Sm22a-Cre* and *Twist1^+/–^* mice at P17 and sacrificed them 1 hour later. Tracer accumulation was seen where hotspots are typically located, suggesting that they are already present by the time of weaning (Figure 3A). In CS mice, flow to perisinusoidal dura was reduced (Figure 3, A and C). In 2–4-month-old controls, tracer was also localized to branched hotspots along the TVS and sinus confluence (Figure 3B). In CS animals, we found less tracer in perisinusoidal dura surrounding dorsal and basal MLVs and less accumulation at hotspots (Figure 3, B and C, Supplemental Figure 2, B and C, see also Supplemental Figure 1H for orientation). These results suggest limiting CSF access to perisinusoidal dura may affect the growth and sprouting of MLVs.

We previously showed that *Twist1^fl/fl^:Sm22a-Cre* homozygous mice had less drainage of CSF macromolecules to the dCLNs (22). We asked whether drainage to the dCLNs was similarly reduced in 2–4-month-old *Twist1^+/FLX^:Sm22a-Cre*, *Twist1^+/–^*, and *Fgfr2^+/S252W^:Sm22a-Cre* mice. We injected 45 kDa ovalbumin tracer into the cisterna magna and sacrificed mice 1 hour later. The dCLNs showed less tracer in CS mice (Figure 3, D and E). Notably, tracer deposition in the dCLNs and perisinusoidal dura was normal in *Twist1^+/–^* mice without suture fusion, consistent with normal levels of ICP and intact MLV networks (Supplemental Figure 2, B–F). These results prompted us to reassess our original findings in homozygous *Twist1^fl/fl^:Sm22a-Cre* mice (22). Like *Twist1^+/FLX^:Sm22a-Cre* and *Twist1^+/–^* mice, ICP was increased and there was less tracer in perisinusoidal dura (Supplemental Figure 4, A–C). This indicates that pathological changes to MLVs associate with reduced access to CSF and less macromolecule drainage to the dCLNs across different genetic CS models. Overall, these changes were generally more severe in *Twist1^fl/fl^:Sm22a-Cre* mice, presumably due to the additive effects that insults from the surrounding environment exerted on top of flow deficits.

### MLVs can access CSF via flow through the cerebellar tentorium.

**Figure 4:**
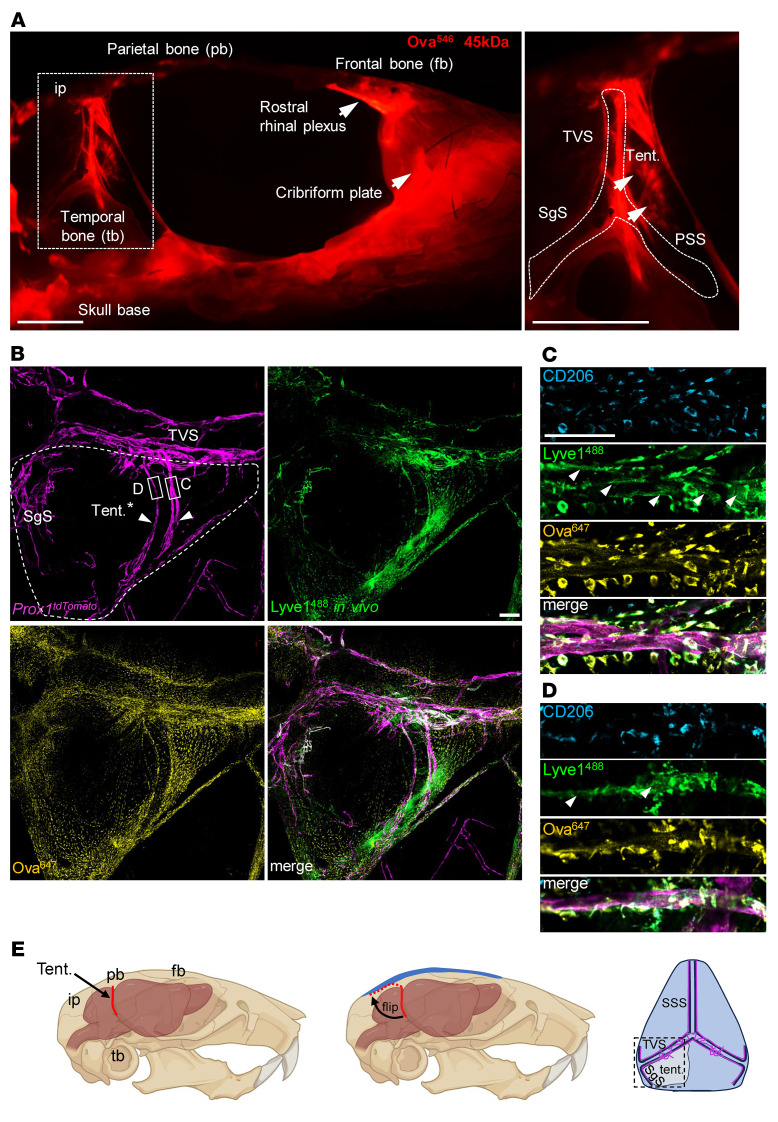
MLVs can access CSF via flow through the cerebellar tentorium. (**A**) Adult skull hemidissected along the dorsal midline. Following injection of 45 kDa ovalbumin tracer into the cisterna magna, tracer is seen draining through the cribriform plate and the skull base. Comet-like streaks of tracer are also seen in the cerebellar tentorium (tent.), as demarcated by arrowheads in the magnified boxed image on right. (**B**) Compressed z-stacks of a dural flatmount showing MLVs labeled with *Prox1-tdTomato* traversing the tentorium (denoted by arrowheads). These vessels form extensions with lymphatic hotspots located along the transverse sinus (TVS). Injecting conjugated Lyve-1^488^ antibody (green) or 45 kDa ovalbumin tracer (gold) into CSF via the cisterna magna shows labeling in the tentorium and also MLVs located at hotspots along the TVS and sigmoid sinus (SgS). (**C** and **D**) Corresponding magnified images of boxed regions of interest in panel B. MLVs traversing the tentorium show staining for Lyve-1^488^ (in vivo, green, arrowheads) and 45 kDa ovalbumin. Neighboring CD206^+^ dural macrophages are also labeled by the antibody and engulf the tracer. (**E**) Schematic overviews illustrating the orientation of the tentorium in panel **B**. The tentorium, a dural inflection that separates the cortex from the cerebellum and brainstem, was carefully preserved, flipped backward during coverslipping, and flat mounted on dura that underlies the interparietal bone (ip) for visualization purposes (dotted red outline). pb, parietal bone; fb, frontal bone; tb, temporal bone; SSS, superior sagittal sinus. Scale bars: 2.5 mm (**A**); 200 μm (**B**); and 100 μm (**C**).

Following tracer injection into the cisterna magna of control mice, we hemidissected the skull along the dorsal midline to visualize outflow routes. As previously reported, tracer was detected at the cribriform plate and along the skull base where MLVs line the dural sheaths of exiting nerves (28–30). Tracer was also concentrated around MLVs at the rostral rhinal venous plexus. Interestingly, streaks of tracer were evident in the cerebellar tentorium, and these appeared to terminate at dorsal and basal hotspots (Figure 4A). We did not see tracer deposition in dura where MLVs grow alongside the middle meningeal arteries, consistent with previous reports (5) (Supplemental Figure 1F). This is notable, considering that arterial MLVs are not affected in CS mice, unlike vessels that grow along the venous sinuses with access to CSF. Through careful preservation of the cerebellar tentorium, we observed lymphatic vessels traversing regions where CSF tracer accumulated. These vessels appeared to terminate at lymphatic hotspots located along the TVS (Figure 4, B and E). Some tracer was found inside these vessels, whereas most was engulfed by dural macrophages (Figure 4, C and D). We also examined uptake by injecting fluorescently conjugated Lyve-1 antibody into CSF. Labeling was detected in basal and dorsal vessels (especially at hotspots), and some labeling was apparent in vessels traversing the tentorium 1-hour after injection (Figure 4, C and D). These findings are intriguing because the mechanisms by which MLVs access CSF are unclear, and it has been speculated that there may be regionalized breaks in the barrier, allowing CSF to exit (31). Thus, barrier breaks may be present in the tentorium, allowing MLVs to access CSF.

### CS affects CSF circulation and influx of macromolecules into the brain.

**Figure 5:**
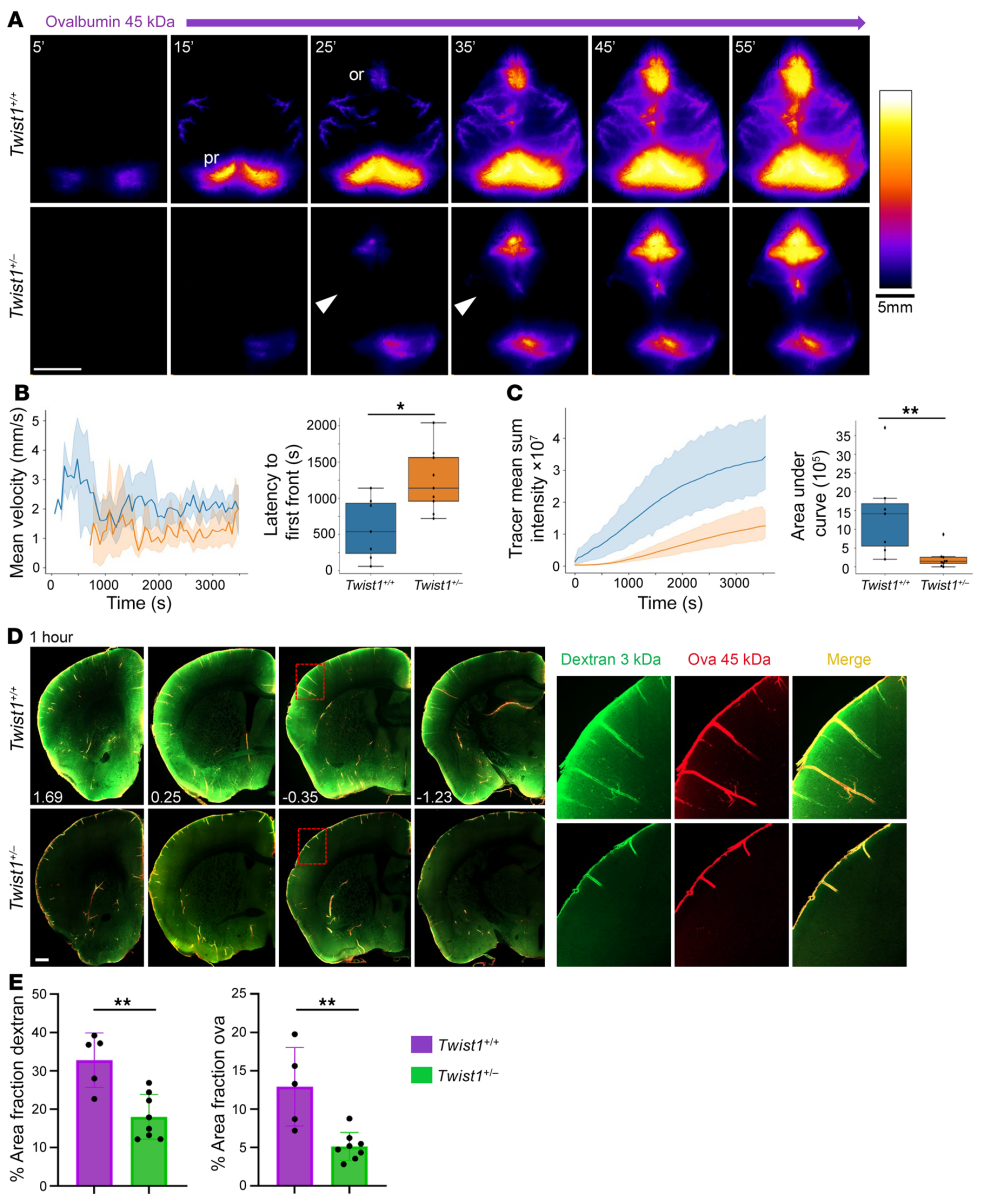
CS affects CSF flow and the perfusion of CSF macromolecules into the brain. (**A**) Representative images taken during transcranial live imaging of a 45 kDa ovalbumin tracer injected into the cisterna magna of young adult mice. Compared with unaffected littermates (top panels), *Twist1^+/–^* mice with CS (bottom panels) show a delay in the appearance of CSF tracer along preferred dorsal pathways (**B**). Throughout 1-hour imaging sessions, the mean sum intensity of tracer was reduced (**C**), especially in the pineal recess and paravascular spaces surrounding penetrating pial arteries (arrowheads) [*Twist1^+/+^* (*n* = 7); *Twist1^+/–^* (*n* = 9)]. (**D**) Perfusion of CSF macromolecules into the brain is reduced in *Twist1^+/–^* mice. Magnified boxed images on the right show that tracer labeling along penetrating arteries is shallower in *Twist1^+/–^* mice compared with unaffected littermates. (**E**) Quantification of percent area fraction of 3 kDa dextran and 45 kDa ovalbumin in brain slices [*Twist1^+/+^* (*n* = 5); *Twist1^+/–^* (*n* = 8)]. *P ≤ 0.05, **P ≤ 0.01 (**B** and **C**) Mann-Whitney U test with Bonferroni correction. (**E**) 2-tailed unpaired *t* test. Scale bars: 5 mm (**A**); 500 μm (**D**).

Genetic, pharmacological, and surgical methods that ablate MLVs or block drainage to the dCLNs diminish paravascular (i.e., glymphatic) influx of CSF macromolecules into the brain (17). This prompted us to investigate if CSF circulation and brain perfusion were affected in CS mice. We coinjected 3 kDa dextran and 45 kDA ovalbumin tracers into the cisterna magna and visualized tracer flow by transcranial live imaging through the skull (32). In unaffected controls, tracer preferentially flowed along previously reported pathways (4, 33). Tracer was first detected along dorsal routes, with accumulation seen in the pineal recess and tentorium where MLVs reside. Approximately 15–20 minutes later, tracer in basal circulation pathways began to pool in the olfactory recess. Around this time, tracer was also seen in paravascular spaces surrounding penetrating pial arteries, indicative of brain perfusion by CSF (34) (Figure 5A). In adult *Twist1^+/–^* mice, there was typically a 5–10-minute delay in the appearance of tracer flow along dorsal routes, with less signal seen in the pineal recess and along the dorsal cortex (Figure 5, A–C). Basal flow was less affected in some mice than others, as judged by pooling in the olfactory recess. Labeling along penetrating pial arteries, especially along more dorsal regions of the cortex, was also diminished (Figure 5A). Thus, CS appeared to perturb CSF circulation along preferred dorsal pathways.

Postmortem examination of brain tissue from *Twist1^+/–^* CS mice showed a significant reduction of 3 kDa dextran and 45 kDa ovalbumin tracers (Figure 5, D and E). Depth of tracer labeling along penetrating pial arterioles was shallower than in unaffected littermates. Similar findings were also seen in postmortem brains from *Fgfr2^+/S252W^:Sm22a-Cre* animals (Supplemental Figure 5, A and B). By contrast, brain-CSF perfusion was unaffected in *Twist1^+/–^* mice without suture fusion, suggesting deficits in flow and brain-CSF perfusion are linked to synostosis (Supplemental Figure 2, G and H). These results suggest that CS impedes brain-CSF perfusion and the clearance of waste and macromolecules from the brain.

### AQP4 polarization to glial endfeet is reduced along penetrating cortical vessels in CS.

Paravascular influx of CSF and glymphatic waste exchange are dependent upon aquaporin-4 (AQP4), a selectively permeable water channel that is polarized to glial endfeet (34). Injecting tracers of various sizes into CSF of AQP4-KO mice results in a significant loss of influx and penetration beneath the brain surface (35). We therefore asked whether the polarization of AQP4 to glial endfeet was affected in CS. We first looked at P17 cortices, at which time AQP4 polarization to glial endfeet is nearing completion (4). We found a subtle, albeit significant, reduction along large caliber vessels (Supplemental Figure 6, A and B). Biochemical fractionation of blood vessels with tethered glial endfeet from whole brain tissue also revealed a reduction in AQP4 protein (Supplemental Figure 6, C and D). In adult animals with CS, however, we did not detect obvious differences in AQP4 immunolabeling, suggesting that polarization to glial endfeet was delayed at earlier time points. Nonetheless, colabeling with lectin and AQP4 still showed a reduction along larger caliber vessels (Supplemental Figure 6, E and F). Thus, CS appeared to delay and/or affect the polarization of AQP4 to glial endfeet abutting large cortical vessels, which may exert subtle effects on brain-CSF perfusion. However, this did not appear to be a major factor affecting perfusion in our models. Instead, changes to preferred CSF circulation pathways and/or insults to MLVs and drainage to the dCLNs appear more likely (17).

### CS exacerbates amyloid-β pathology in 5xFAD mice.

**Figure 6:**
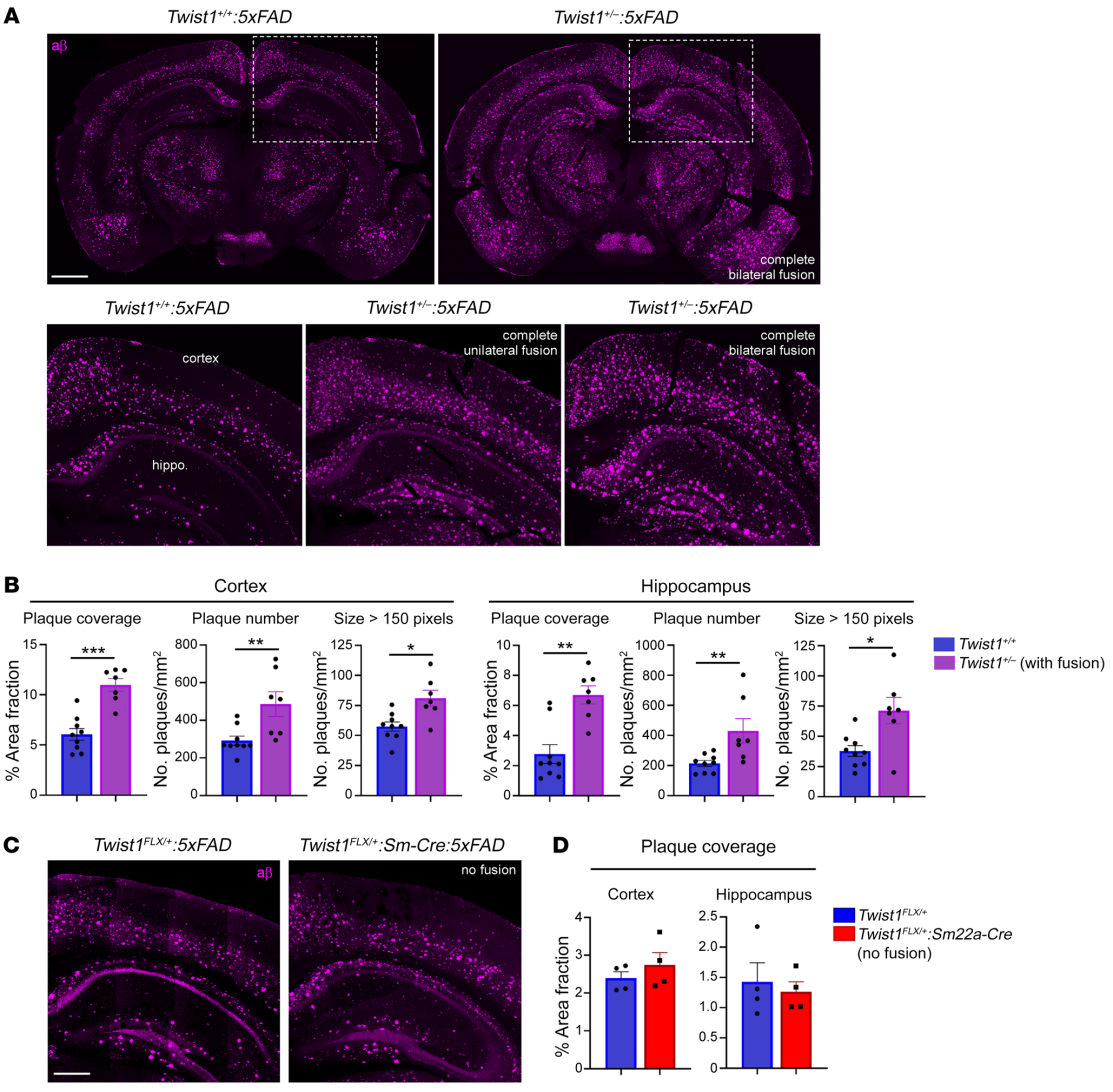
CS exacerbates amyloid-β pathology in 5xFAD transgenic mice. (**A**) Representative images of amyloid-β in the cortex and hippocampus of 5–6-month-old 5xFAD mice with CS (top right and bottom panels) and without CS (top left, bottom left magnified image). (**B**) Quantification of amyloid plaque coverage, number, and size in the cortex and hippocampus [*Twist1^+/+^:5xFAD* (*n* = 9); *Twist1^+/–^:5xFAD* (*n* = 7)]. (**C**) Representative images of amyloid-β in the cortex and hippocampus of 5-month-old *5xFAD* and *Twist1^FLX/+^:Sm22a-Cre:5xFAD* mice without suture fusion. (**D**) Quantification of percent area fraction of amyloid-β in the cortex and hippocampus. No differences are seen in amyloid-β coverage [*Twist1^FLX/+^* and *Twist1^FLX/+^:Sm22a-Cre* (*n* = 4)]. *P ≤ 0.05, **P ≤ 0.01, ***P ≤ 0.001 Mann-Whitney U test. Scale bars: 1 mm (**A**); 500 μm (**C**).

Inhibiting MLV drainage impairs brain-CSF perfusion and macromolecule clearance from the brain (13, 17, 28). Also, ablation of MLVs using Visudyne aggravates amyloid-β pathology in *5xFAD* mice, which express 5 human mutations found in AD and develop amyloid-β plaques (17). We investigated whether loss of MLV drainage and brain-CSF perfusion in CS exacerbated amyloid-β deposition in *Twist1^+/–^:5xFAD* mice. In 5–6-month-old *Twist1^+/–^:5xFAD* mice, there was a significant increase in area coverage, and number and size of amyloid-β plaques in both the cortex and hippocampus compared with *5xFAD* mice without CS (Figure 6, A and B). Immunostaining for GFAP and IBA1 in *Twist1^+/–^* CS mice revealed normal coverage and also microglia morphology, suggesting no changes to astro- and microgliosis under steady state (Supplemental Figure 7, A–C). Notably, suture fusion was very mild or nonexistent in *Twist1^+/FLX^:Sm22a-Cre:5xFAD* mice, presumably due to background strain differences. We did not see a significant increase in area coverage, number of amyloid-β plaques, and aggregate size in either the cortex or hippocampus (Figure 6, C and D). This suggests CS and corresponding deficits in brain-CSF perfusion and MLV drainage are linked to increased amyloid-β buildup in *Twist1^+/–^:5xFAD* mice.

### Piezo1 activation reduces ICP and improves brain-CSF perfusion and meningeal lymphatic functions in CS mice.

**Figure 7:**
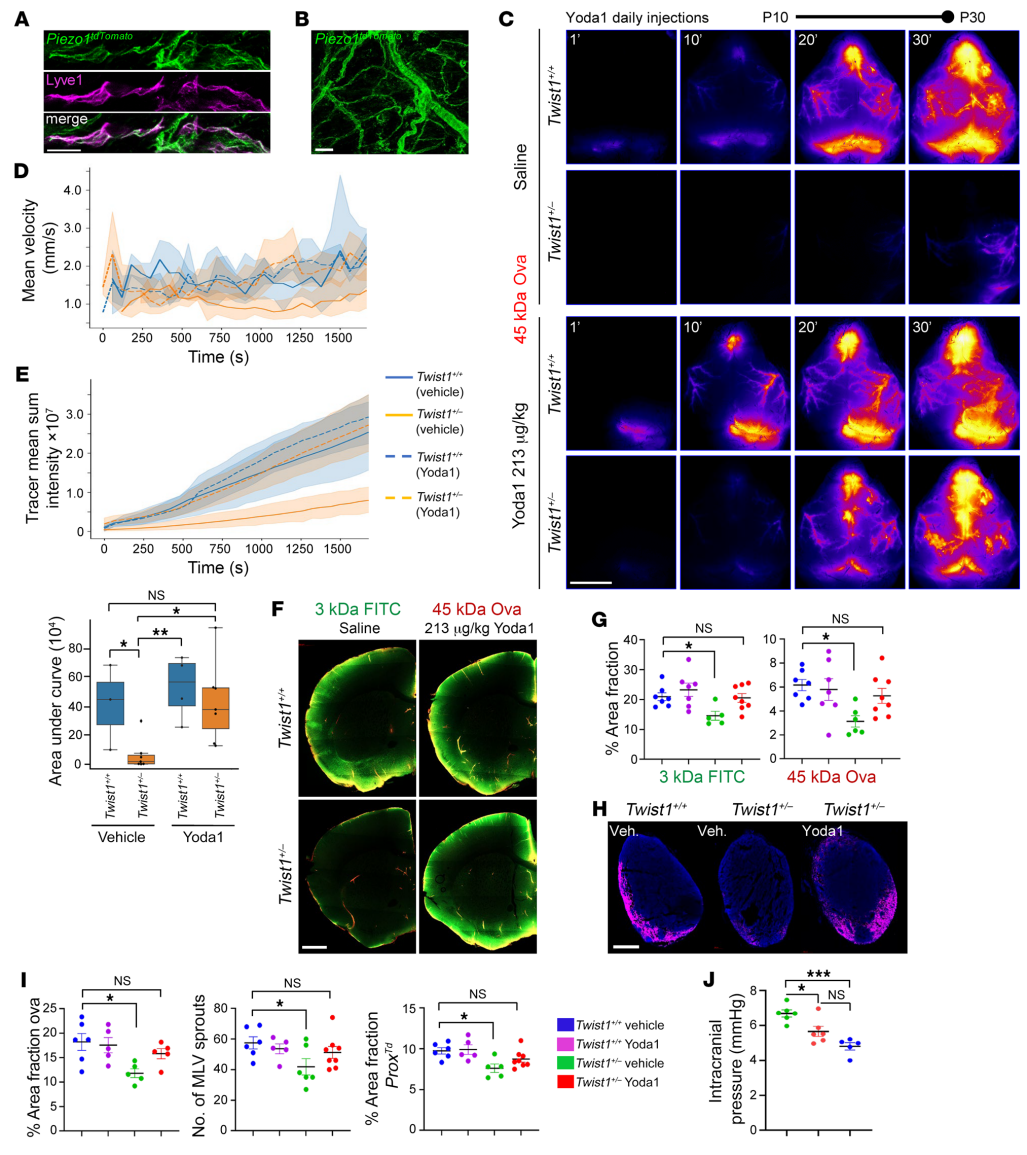
Piezo1 activation helps restore MLV functions and brain-CSF perfusion in CS. (**A**) *Piezo1^tdTomato^* expression (depicted in green) is detected in MLVs colabeled with Lyve-1. (**B**) *Piezo1^tdTomato^* expression is also found in small and large caliber meningeal blood vessels. (**C**) Representative transcranial time-lapse images from juvenile P30 mice treated daily with saline vehicle (top 2 panels) or 213 μg/kg Yoda1 (bottom 2 panels). Yoda1 helps restore CSF flow along preferred circulation pathways in CS mice. (**D**) Graphs depicting tracer mean velocity and (**E**) mean sum intensity in *Twist1^+/+^* and *Twist1^+/–^* mice treated with vehicle or Yoda1 [*Twist1^+/+^* vehicle (*n* = 3); *Twist1^+/+^* Yoda1 (*n* = 4); *Twist1^+/–^* vehicle (*n* = 6); *Twist1^+/–^* Yoda1 (*n* = 7)]. (**F**) Representative brain sections from juvenile P30 mice treated daily with saline vehicle (left panels) or 213 μg/kg Yoda1 (right panels). Perfusion of CSF macromolecules into the brain is significantly improved in *Twist1^+/–^* mice treated with Yoda1. (**G**) Quantification of percent area fraction of 3 kDa dextran and 45 kDa ovalbumin in brain slices [*Twist1^+/+^* vehicle (*n* = 7); *Twist1^+/+^* Yoda1 (*n* = 7); *Twist1^+/–^* vehicle (*n* = 5); *Twist1^+/–^* Yoda1 (*n* = 8)]. (**H**) Representative dCLN sections from juvenile P30 mice treated daily with saline vehicle or with 213 μg/kg Yoda1. (**I**) Yoda1 treatment helps restore ovalbumin drainage to the dCLNs [*Twist1^+/+^* vehicle (*n* = 6); *Twist1^+/+^* Yoda1 (*n* = 5); *Twist1^+/–^* vehicle (*n* = 5); *Twist1^+/–^* Yoda1 (*n* = 5)], the number of MLV sprouts [*Twist1^+/+^* vehicle (*n* = 6); *Twist1^+/+^* Yoda1 (*n* = 5); *Twist1^+/–^* vehicle (*n* = 6); *Twist1^+/–^* Yoda1 (*n* = 8)] and percent area coverage of *Prox1-tdTomato* signal in dura [*Twist1^+/+^* vehicle (*n* = 6); *Twist1^+/+^* Yoda1 (*n* = 5); *Twist1^+/–^* vehicle (*n* = 6); *Twist1^+/–^* Yoda1 (*n* = 8)]. (**J**) Yoda1 significantly reduces ICP in young adult *Twist1^+/–^* mice with unilateral or bilateral CS [*Twist1^+/–^* vehicle (*n* = 6); *Twist1^+/–^* Yoda1 (*n* = 6); *Twist1^+/+^* vehicle (*n* = 5)]. *P ≤ 0.05, **P ≤ 0.01, ***P ≤ 0.001. 1-way ANOVA with Bejamini-Hochberg correction (**E**), Dunnett’s (**G** and **I**), and Tukey’s multiple comparison test (**J**). Scale bars: 20 μm (**A**); 50 μm (**B**); 5 mm (**C**); 1 mm (**F**); and 200 μm (**H**).

Raised ICP in CS may result from several factors, including craniocerebral disproportion and/or venous hypertension coupled with changes to cerebral blood flow (25). Piezo1, a mechanosensitive ion channel expressed in vascular endothelial cells and smooth muscle, is suggested to control cerebral capillary blood flow, vascular tone, and blood pressure (36, 37). This implies it may be involved in the regulation of ICP. Studies have also indicated a role for Piezo1 in the regulation of intraocular pressure (38). In addition, force exerted by unidirectional laminar flow activates Piezo1-mediated mechanotransduction signaling in lymphatic vessels to control sprouting, expansion, and long-term maintenance (7). Given that we detected Piezo1 expression in meningeal blood vessels and perisinusoidal MLVs using *Piezo1^tdTomato^* reporter mice (Figure 7, A and B), we tested whether activating Piezo1 could reduce the levels of ICP and/or help restore brain-CSF perfusion and meningeal lymphatic functions in *Twist1^+/–^* CS mice.

Yoda1 is a small molecule agonist that has high specificity and affinity for Piezo1, lowering the mechanical threshold for channel activation (39). We treated *Twist1^+/–^* mice daily, starting at P10 until P30, with intraperitoneal injections of Yoda1. We next tested if this treatment could help restore brain-CSF perfusion in CS mice. We injected 3 kDa dextran and 45 kDa ovalbumin tracers into CSF and sacrificed mice 30 minutes later. In juvenile *Twist1^+/–^*:*Prox1-tdTomato* mice treated with saline vehicles, CSF circulation along dorsal pathways was perturbed, similar to findings in adults (Figure 7, C–E). In postmortem brain tissue, tracer deposition was significantly reduced (Figure 7, F and G). Strikingly, in *Twist1^+/–^*:*Prox1-tdTomato* mice treated with Yoda1, CSF flow along dorsal pathways was restored (Figure 7, C–E). Furthermore, brain-CSF perfusion was also improved, as the differences between *Twist1^+/–^*:*Prox1-tdTomato* mice treated with Yoda1 and unaffected littermates treated with either saline vehicles or Yoda1 were no longer significant (Figure 7, F and G). We still, however, found subtle decreases in AQP4 at glial endfeet abutting large caliber vessels, which may have prevented a more complete rescue in some animals (Supplemental Figure 6G).

Next, we tested if macromolecule drainage to the dCLNs also improved. Owing to random selection of animals at P10 when suture fusion is harder to gauge because it is an ongoing process (40), MLV phenotypes in P30 *Twist1^+/–^*:*Prox1-tdTomato* mice treated with saline vehicles were slightly less severe due to milder suture fusion (partial unilateral) in this cohort. Nonetheless, MLV sprouting and coverage along the TVS, plus drainage to the dCLNs, was improved in *Twist1^+/–^*:*Prox1-tdTomato* mice treated with Yoda1, as differences were no longer significant compared to unaffected controls treated with vehicle or Yoda1 (Figure 7, H and I). Thus, activating Piezo1 with small molecule agonists can help rescue insults to meningeal lymphatic functions and brain-CSF perfusion in mice with CS.

We next tested whether improvement to brain-CSF perfusion and meningeal lymphatic functions could result from reducing the levels of ICP. Treating *Twist1^+/–^* mice that had either unilateral or bilateral fusion with Yoda1 reduced the levels of ICP. At 2 months of age, ICP was significantly lower in Yoda1-treated CS mice compared with CS mice receiving saline vehicle. Further, the levels of ICP in Yoda1-treated CS mice did not show significant differences compared with untreated control mice (Figure 7J). Thus, activating Piezo1 with a small molecule agonist in CS may help rescue insults to CSF flow, MLV drainage, and brain-CSF perfusion, at least in part, by reducing ICP. These findings are notable because they also suggest that raised ICP in CS may be caused, in large part, from effects exerted by the vasculature, CSF, and/or blood flow.

**Figure 8:**
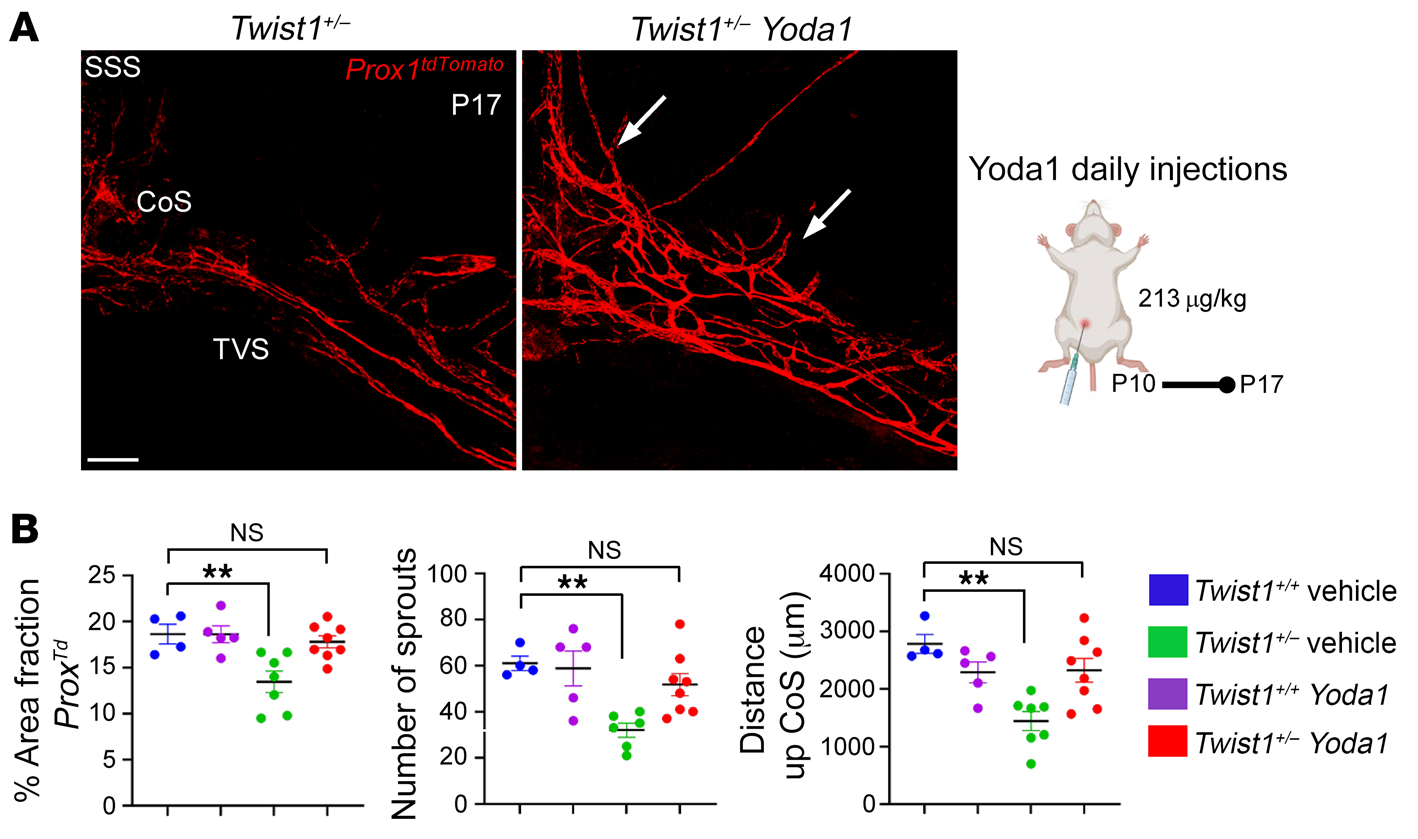
Piezo1 activation restores MLV growth and sprouting in juvenile CS pups. (**A**) Representative images from juvenile P17 mice treated daily with saline vehicle or 213 μg/kg Yoda1. Yoda1 restores MLV growth and sprouting along the transverse sinuses. (**B**) Quantification of percent area fraction of *Prox1-tdTomato* signal, number of sprouts, and distance of growth from the confluence of sinuses [*Twist1^+/+^* vehicle (*n* = 4); *Twist1^+/+^* Yoda1 (*n* = 5); *Twist1^+/–^* vehicle (*n* = 7); *Twist1^+/–^* Yoda1 (*n* = 8)]. SSS, superior sagittal sinus; TVS, transverse sinus; CoS, confluence of sinuses. **P ≤ 0.01, 1-way ANOVA with Dunnett’s multiple comparison test. Scale bar: 200 μm.

Finally, we asked if Piezo1 activation could restore MLV growth and sprouting in CS mice. We treated P10 *Twist1^+/–^* mice and littermate controls with Yoda1 for 7 consecutive days. In P17 *Twist1^+/–^*:*Prox1-tdTomato* mice treated with saline vehicle, we saw less MLV sprouts and growth along the TVS, confluence, and SSS (Figure 8, A and B). However, meningeal lymphangiogenesis was improved in *Twist1^+/–^*:*Prox1-tdTomato* mice that received Yoda1 (213 μg/kg), as these differences were no longer significant between unaffected littermates treated with saline or those treated with Yoda1 (Figure 8, A and B). Thus, activating Piezo1 signaling in *Twist1^+/–^* CS pups can also rescue impairments to MLV growth and expansion.

### Stimulating Piezo1 in aged animals helps restore meningeal lymphatic functions and brain-CSF perfusion.

**Figure 9:**
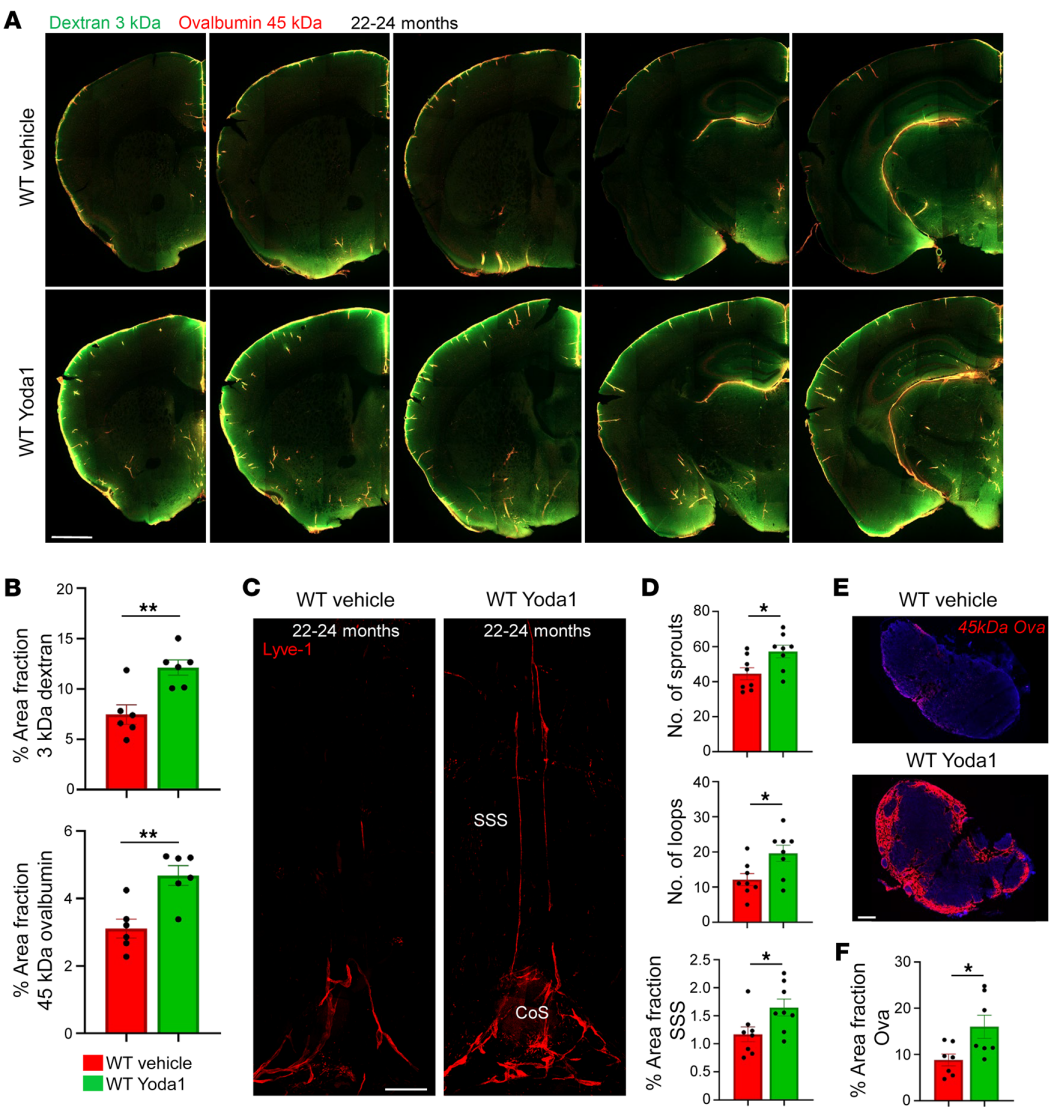
Piezo1 activation helps restore MLV networks and brain-CSF perfusion in aged animals. (**A**) Representative images show that perfusion of CSF macromolecules into the brain is reduced in 22–24-month-old mice treated with saline vehicle. Yoda1 treatment improves brain-CSF perfusion as seen by a significant increase in the amount of 3 kDa dextran and 45 kDa ovalbumin tracer in tissue. (**B**) Quantification of percent area fraction of 3 kDa dextran and 45 kDa ovalbumin in brain slices [WT vehicle and WT Yoda1 (*n* = 6)]. (**C**) Representative images show Yoda1 treatment in 22–24-month-old mice increases MLV coverage along the SSS and the numbers of loops and sprouts along the sinus confluence and proximal transverse sinus. (**D**) Quantification of numbers of sprouts, loops, and percent area fraction of Lyve-1 signal along the SSS [WT vehicle and WT Yoda1 (*n* = 8)]. (**E**) Tracer drainage to the dCLNs is significantly improved in 22–24-month-old mice receiving Yoda1 versus saline vehicle. (**F**) Quantification of percent area fraction of 45 kDa ovalbumin tracer [WT vehicle and WT Yoda1 (*n* = 7)]. SSS, superior sagittal sinus; CoS, confluence of sinuses. *P ≤ 0.05, **P ≤ 0.01, 2-tailed unpaired *t* test. Scale bars: 500 μm (**A** and **C**); 200 μm (**E**).

Pathological changes to MLVs, drainage to the dCLNs, and brain-CSF perfusion in CS mice are reminiscent of findings from aged animals, which have diminished MLV drainage and brain-CSF perfusion compared with young and middle-aged adults. We tested whether stimulating Piezo1 signaling with Yoda1 could also improve MLV drainage and brain-CSF perfusion in mice aged 22–24 months. Yoda1-treated mice showed a significant increase in brain CSF-perfusion, as measured by the amount of tracer in brain tissue (Figure 9, A and B). Furthermore, MLV complexity was increased, as measured by the number of loops and sprouts along the TVS and at the confluence (Figure 9, C and D). Vessel coverage along the SSS was also increased, as was the amount of tracer in the dCLNs (Figure 9, D–F). Thus, stimulating Piezo1 mechanotransduction signaling in aged mice enhanced MLV coverage, drainage, and brain-CSF perfusion.

## Discussion

Pathological changes to MLVs in CS models underscore their utility for elucidating environmental processes that drive meningeal lymphangiogenesis and long-term maintenance of those vessels. In homozygous *Twist1^fl/fl^:Sm22a-Cre* mice, these were previously attributed to loss of growth factor signaling from venous smooth muscle and/or hypoplastic dura (22). However, the venous sinuses and surrounding dura are unaffected in *Twist1^+/–^*, *Twist1^+/FLX^:Sm22a-Cre*, and *Fgfr2^+/S252W^:Sm22a-Cre* mice. Instead, we show that CS causes raised ICP and impedes CSF flow, a consistent finding across all models, including *Twist1^fl/fl^:Sm22a-Cre* mice. In addition, activating Piezo1, a mechanosensitive ion channel, with Yoda1 reduces ICP in *Twist1^+/–^* mice. This is associated with improved MLV growth, drainage, and also brain-CSF perfusion. Thus, our results suggest Piezo1 is involved in the regulation of ICP, and small molecule treatments targeting Piezo1 may be effective for reducing ICP in pathological states. Our results also suggest that raised ICP in CS may be largely vascular and fluid based, as Yoda1 treatment reduced ICP in mice with fused sutures and malformed skulls. Stimulating Piezo1 signaling in other fluid disorders that associate with raised ICP may have similar beneficial effects on lymphatic networks and brain-CSF perfusion.

In addition to reducing ICP, Yoda1 may have direct effects on MLVs. Laminar flow is required for lymphatic growth and maintenance, and it activates Piezo1 mechanotransduction signaling in lymphatic vessels to promote lymphangiogenesis (7, 41, 42). Conditional inactivation of *Piezo1* in embryonic lymphatic endothelial cells inhibits sprouting and expansion, whereas activation of Piezo1 increases lymphatic vessel density and number of vascular tips in 3D cultures (7). Furthermore, *Piezo1* inactivation in postnatal mice causes lymphatic regression in the mesentery and hindlimb, suggesting that laminar flow may also be required for long-term maintenance of MLVs. Thus, Piezo1-mediated mechanotransduction signaling supports lymphatic sprouting, expansion, and maintenance — all of which are affected in CS mice. Another intriguing possibility is that CSF provides mechanical force to facilitate MLV sprouting and expansion. Limiting MLV access to CSF may impair their development and long-term maintenance in CS, potentially via perturbing mechanotransduction signaling. Notably, MLVs lining meningeal arteries are not affected in CS mice and these vessels do not appear to have access to CSF (5). Thus, improvement to CSF circulation following reduction of ICP through Yoda1 treatment may, in turn, exert mechanical force that facilitates the restoration of MLV growth and drainage. However, further studies, including the effects of lymphatic-specific KO of Piezo1 on MLVs, are still needed, as this was not directly addressed in the current study. Furthermore, increasing interstitial flow and functional drainage can stimulate VEGF-C expression, VEGFR3 activation, and cell proliferation via integrin-dependent interactions between lymphatic endothelial cells and the extracellular matrix (8). Thus, it remains possible that multiple signaling pathways are affected in CS mice, impinging upon lymphatic vessels. This will require further investigation.

Our data in aged mice also suggest CSF may support long-term maintenance of MLVs. The natural decline of CSF flow and turnover as animals age may cause MLVs to regress or undergo pathological changes indicative of functional impairments to fluid drainage. Similar to peripheral lymphatics, MLVs require VEGF-C/VEGFR3 signaling for growth and expansion (2). Once networks have formed, however, VEGF-C/VEGFR3 signaling is still required for survival and long-term maintenance, particularly for dorsal vessels along the TVS and SSS that are sensitive to VEGF-C (2). Similarly, our data suggest that Piezo1 mechanotransduction signaling promotes MLV sprouting, expansion, and also long-term maintenance, presumably due to direct effects on vessels versus ICP, which is not known to be affected in aged but otherwise healthy mice. The gradual decline of CSF flow, turnover, and drainage with ageing, or changes to preferred circulation pathways in pathological states, may inhibit both VEGF-C/VEGFR3 signaling and Piezo1 activation. This may cause dorsal networks to regress and basal networks to undergo lymphedematous-like changes. Considering that adenoviral-mediated expression of VEGF-C can improve MLV functions and help restore brain-CSF perfusion in aged mice — similar to the effects of Yoda1 — combined treatments may provide greater beneficial effects for the long-term functional maintenance of MLVs and brain-CSF perfusion (17).

The demise of brain waste clearance with ageing or in pathological states is proposed to be an underlying risk factor for dementia (43, 44). Brain-CSF perfusion is most active during sleep (45, 46), and sleep disorders are common in the elderly. As mice age, dorsal MLVs regress, whereas basal MLVs become hyperplastic, concurrent with the decline of brain-CSF perfusion (16, 17, 43). As such, the demise of brain waste clearance in CS appears to have early onset. Initially, the growth and sprouting of MLVs is affected in juveniles, and dorsal MLV networks are typically hypoplastic in young adults. As mice reach middle age, dorsal MLVs continue to regress and basal vessels become hyperplastic. The distinct pathological changes to dorsal versus basal MLVs in CS may reflect exposure to different flow conditions, which also change with ageing (14). Furthermore, the decline of CSF flow and turnover with ageing may be comparable to loss of normal CSF circulation seen in CS mice. These data suggest that impairments to CSF flow in pathological conditions such as CS may accelerate the deterioration of MLVs and brain waste clearance. Thus, genetic models for CS have the power to elucidate not only how these systems develop, but also factors that are required for long-term maintenance.

Optimal brain-CSF perfusion requires AQP4 water channels at glial endfeet, which line paravascular spaces surrounding blood vessels (35). Following MLV ablation using Visudyne, brain-CSF perfusion is significantly reduced, but without changes to AQP4 localization or expression (17). In juvenile CS mice, we see an initial delay in AQP4 polarization to endfeet. We do not see widespread changes to AQP4 expression or localization in adults, however, except for subtle reductions at glial endfeet along large caliber vessels. Considering that AQP4-KO mice show only a 30%–50% reduction in the perfusion of CSF macromolecules into the brain, the changes we detect in CS mice are unlikely to significantly affect brain-CSF perfusion. Interestingly, as mice age, AQP4 is lost at the glial endfeet that line penetrating pial arterioles (43), in line with our observations that brain waste clearance systems show signs of precocious ageing in CS. Overall, our data agrees with previous reports that suggest brain-CSF perfusion is integrated with lymphatic drainage in the dura (17). Ageing-related or pathological conditions that hinder drainage to the dCLNs are likely to have deleterious consequences for brain-CSF perfusion and waste clearance. It is also important to note that brain-CSF perfusion depends on cerebral arterial pulsatility, which declines in aged mice (43). Improved brain-CSF perfusion in aged mice following Yoda1 treatment may also involve changes to arterial pulsatility and blood flow, an avenue for future investigation in both aged mice and CS.

Deficits in MLV drainage and brain-CSF perfusion are predicted to exacerbate amyloid-β pathology in *Twist1^+/–^*:*5xFAD* models. Our findings agree with previous studies that show that restricting MLV drainage and brain-CSF perfusion increases amyloid-β burden in *5xFAD* mice (17). Further, ablating MLVs leads to worse outcomes and greater deposition of amyloid-β in *5xFAD* mice treated with anti–amyloid-β passive immunotherapy, whereas increasing MLV functions with exogenous VEGF-C improves efficacy (18). Interestingly, recent findings in *5xFAD* mice show that Yoda1 can increase amyloid-β phagocytosis via effects on microglia (47, 48). It remains to be seen what effects Yoda1 has on amyloid-β burden in *Twist1^+/–^*:*5xFAD* mice. Our results suggest that Yoda1 may act to reduce amyloid-β burden in *Twist1^+/–^*:*5xFAD* mice through combined effects on microglia and mechanosensing, as well as improving MLV drainage and brain-CSF perfusion. Moreover, Yoda1 may provide similar benefits to counteract the buildup of neurotoxins, such as tau and amyloid-β, with ageing and in response to brain injury.

Deficits in MLV drainage and brain-CSF perfusion in CS show how skull expansion is integrated with the development of brain waste clearance systems. The human skull expands rapidly after birth. Concurrently, the brain nearly triples in size and, along with increasing volumes of blood and CSF within the fixed confines of the skull, this causes ICP to quickly rise (49). ICP needs to be controlled in order to maintain proper fluid dynamics in the head. This suggests that skull expansion, via effects on ICP and CSF, exerts noncell autonomous control over MLV development. The growth and remodeling of MLVs also mirrors the basal-dorsal establishment of CSF circulation pathways while the skull expands (4). We propose that impediments to CSF flow caused by CS may potentially affect MLVs by restricting access to CSF. Notably, arterial MLVs are not affected in CS and we do not detect tracer around these vessels, suggesting that their development and functions are less dependent on CSF and perhaps more related to draining dural ISF. In addition, polarization of AQP4 water channels to glial endfeet also mirrors the establishment of CSF circulation pathways (4). It is therefore possible that the development of the glymphatic and meningeal lymphatic systems are interconnected via shared dependency on CSF flow and mechanical forces that are influenced by skull growth. Thus, failure to maintain normal CSF flow in conditions like CS or hydrocephalus is likely to affect MLV development and brain-CSF perfusion, potentially leading to accumulation of waste and neurotoxins.

Raised ICP is associated with neurological and cognitive dysfunction in CS (24). Surgical interventions to alleviate raised ICP include removing fused sutures and invasive craniotomies to remodel and expand the skull. However, these procedures sometimes fail to maintain normal levels of ICP. For example, upward of 40% of individuals with *TWIST1* mutations may still experience raised ICP following surgery (50). A recent study showed that engraftment of sutural progenitor cells into affected sutures of *Twist1^+/–^* mice prevented fusion and improved skull growth, while reducing ICP and restoring cognitive functions (26). Our results now suggest deficits in MLV drainage, CNS waste clearance, or perhaps altered neuroimmune surveillance may contribute to cognitive impairment in CS. Further, those who suffer from CS and/or chronically raised ICP may have increased risk for neurodegenerative disease. Our findings showing that Yoda1 can reduce ICP and help restore MLV functions and brain-CSF perfusion in CS therefore have important clinical implications. Similar treatments may hold promise in humans, while also providing what we believe to be novel avenues to restore brain-CSF perfusion and stave off the toxic effects of waste on brain health and function with ageing.

## Methods

### Mice.

Mice were maintained on a regular light/dark cycle, with experiments conducted during the light cycle. We utilized the following transgenic mice: *Twist1^FLX^* (University of North Carolina (UNC); RRID:MMRRC_016842-UNC), *Prox1^tdTomato^* (University of California, Davis; RRID:MMRRC_036531-UCD), *Sm22a-cre* (The Jackson Laboratory; RRID:IMSR_JAX:017491), *Sox2-cre* (The Jackson Laboratory; RRID:IMSR_JAX:008454), *Fgfr2^S252W^* (gift from Ethylin Jabs, Icahn School of Medicine, Mt. Sinai, New York, New York, USA), *5xFAD* (The Jackson Laboratory; RRID:MMRRC_034840-JAX), and *Piezo1^tm1.1Apat/j^* (The Jackson Laboratory; RRID:IMSR_JAX:029214). Male and female mice were included in all experiments, except for aged cohorts receiving saline or Yoda1 treatments, which consisted of only female animals (The Jackson Laboratory; RRID:IMSR_JAX:000664). Animals were bred on a C57Bl/6J or mixed genetic background (C57Bl/6J:CD1). *Twist1*^+/–^ mice were produced by breeding *Twist1^FLX/+^* males with female Sox2-cre mice for germline recombination. Animal ages in this study ranged from postnatal days (P) 17 and 30 to 2–4 months, 6–8 months, 10–14 months, or 22–24 months. Mutant mice at P17 were included if some synostosis was evident. Adult animals were included if they exhibited full unilateral or near-complete/full bilateral coronal synostosis, as judged by inspection under a stereomicroscope. Adult animals with unilateral fusion were also included if greater than 50% of the suture was fused and the skull was dysmorphic, as shown in Figure 1B. Aged animals were purchased from The Jackson Laboratory at 96 weeks and were allowed to mature to 22–24 months in our colony.

### Antibodies.

The following antibodies were used: rabbit anti-Lyve1 (1:500, Abcam ab14917 RRID:AB_301509), rabbit anti-RFP (1:1,500, Rockland, 600-401-379, RRID:AB_2209751), rabbit anti-amyloid β (1:400, Cell Signaling, D54D2, RRID:AB_2797642), rabbit anti-Aqp4 (1:500, Invitrogen PA5-78812, RRID:AB_2745928), rabbit Mouse anti-Crabp2 (1:100, Sigma-Aldrich, MAB5488, RRID:AB_2085470), mouse anti-smooth muscle actin (1:500 Sigma-Aldrich, C6198, RRID:AB_476856), GS-Lectin IB4 488 and 649 (1:100, Vector Biolabs, no. B-1205-.5 and no. DL-1208-.5), chicken anti-IBA1 (1:500, Aves, IBA1-0100, RRID:AB_2910556), and mouse anti-GFAP (1:500, Santa Cruz, SC33673).

### IHC.

After skull decalcification, tissue underwent overnight primary antibody staining in a solution of 5% NGS (Sigma-Aldrich) in PBS with 0.3% Triton. Secondary antibodies (1:1,000 dilution) in PBS with 5% NGS and 0.3% Triton were applied at room temperature for 1 hour. Secondary antibodies included donkey anti-chicken Alexa Fluor 488, Jackson IR 703-545-155; goat anti-rabbit Alexa Fluor 488, Jackson IR 111-545-144; goat anti-rabbit Cy3, Jackson IR 111-165-144; goat anti-rabbit Alexa Fluor 647, Jackson IR 111-605-144; goat anti-mouse Alexa Fluor 488, Jackson IR 115-545-166; goat anti-rat Alexa Fluor 647, Jackson IR 112-605-167; and goat anti-rat DyLight 405, Jackson IR 112-475-167. For AQP4 staining, 100 mm brain sections were incubated in Dent’s Fix (80% MeOH [VWR], 20% DMSO) overnight at 4°C before primary antibody staining. Piezo1 labeling in *Piezo1^tdTomato^* mice followed established protocols (51). Mice were perfused with heparin-containing PBS (heparin from Sigma-Aldrich), the dura dissected, and fixed in 4% PFA for 20 minutes. Tissue was quenched in 20 mM glycine and 75 mM ammonium chloride (both from Sigma-Aldrich0 with 1% Triton X-100 (Thermo Fisher Scientific) in PBS for 20 minutes, washed, and blocked (0.6% bovine skin gelatin [Sigma-Aldrich], 0.05% saponin in PBS with 5% normal donkey serum[Sigma-Aldrich]) for an hour at room temperature. Staining with anti-rabbit RFP antibody and anti-rat Lyve-1 occurred overnight at 4°C in blocking buffer without serum. After washing, the tissue was stained with secondary antibodies and flat-mounted for imaging.

### Dorsal and basal skull flat mounts.

For dorsal skull flat mounts, dorsal craniotomies were performed by incising at the foramen magnum and using curved-tip spring scissors to radially cut around the base to the orbits. A final incision was made between the orbits to free the skull cap with attached meninges. Skull caps were postfixed in 4% PFA (Sigma-Aldrich) overnight at 4°C with agitation. After fixation, they were immersed in 3% Hydrogen Peroxide for 24 hours at 4°C, followed by overnight Dent’s Fix (80% MeOH, 20% DMSO [Sigma-Aldrich]). Decalcification with 14% EDTA [Boston Bioproducts] took 3–5 days, with solution changes every other day until the skull became malleable. Dorsal skull preparations were trimmed along the TVS and SSS, removing excess bone, before cover slipping the tissue with 600 μL of mounting media.

### Skull 3D X-Ray Microscopy (computed tomography).

Animals were euthanized via transcardial perfusion with 4% PFA. Heads were decapitated and postfixed in 4% PFA overnight. Hair and skin were removed before imaging. Images were captured using a Bruker Skyscan 1272 X-ray microscope under these scan conditions: image pixel size = 13.5 μm, camera = 1,632 columns × 1,092 rows, rotation step = 0.4°, frame averaging = 3, filter = 1 mm Al. Resulting images were reconstructed and converted to dicom format using Skyscan Ctan software. Dicom files were processed in Vivoquant to segment teeth and bone from less dense soft tissue.

### ICP recordings.

Animals aged P17 or between 2–4 months (young adults) were anesthetized with 1% Isoflurane (Covetrus) and 2% oxygen, following a protocol adapted from Da Mesquita et al, 2018 (17). A midline incision extended from below the orbits to the skull apex. The underlying skull was cleaned with sterile saline. A 0.5 mm hole was drilled into the right parietal bone using a dental drill (coordinates: ML + 1.0 mm, AP –1.0 mm from bregma or adjusted for fused sutures). A pressure sensor (Millar SPR100) was inserted at a depth of 1 mm and connected to a PCU-2000 pressure control unit. Pressure readings were averaged over 5 minutes following signal stabilization. To assess Yoda1’s impact on ICP, adult *Twist1*^+/–^ and WT mice received intraperitoneal Yoda1 injections (1 mg/kg) 5 times per week for 4 weeks, starting at 1 month of age. ICP was measured at the end of the 4 weeks (2 months of age). All animals were euthanized after recordings.

### Tracer infusions.

Mice were anesthetized with ketamine/xylazine (100 mg/kg) (ketamine from Covetrus and xylazine from Anased) and underwent a midline incision at the occipital crest. The skin was excised and curved forceps exposed the underlying muscle, which was separated along the midline to access the cisterna magna without inducing tears or bleeding. A mixture of 45 kDa ovalbumin-647 tracer (Molecular Probes, Invitrogen) and 3 kDa fluorescein dextran tracer (Molecular Probes, Invitrogen) in artificial CSF (2 μg/mL and 0.5 μg/mL, respectively) was injected using a 28-gauge needle attached to polyethylene tubing and a Nanomite infusion system (Harvard Apparatus) at a rate of 2.5 μL/min. Animals received supplemental oxygen to stabilize breathing throughout the experiment, and the needle was secured to prevent depressurization. A second dose of ketamine/xylazine (100 mg/kg) was administered approximately 30 minutes after the first, ensuring proper anesthesia, monitored by tail pinches. Upon completion, the needle was removed and mice were euthanized via transcardial perfusion. Tracer infusion experiments lasted 60 minutes, except for P30 Yoda1 experiments, which were conducted for 30 minutes.

### dCLN imaging and quantification.

Animals were perfused with 4% PFA and dCLNs were dissected under fluorescence. Tissue postfixed in 2% PFA for 12 hours was then treated with 30% sucrose (Sigma-Aldrich) and embedded in Neg-50 medium. Sections of 20 μm were cut throughout the entire dCLN length and imaged using a Leica M165FC stereomicroscope equipped with a 1 × Plan objective and DFC7000T camera. Ten 20 μm sections per animal from the dCLNs were equally thresholded and analyzed to calculate the percent area fraction of ovalbumin 45 kDa tracer. Values were averaged for each animal to obtain a single value representing the average percent area fraction of tracer. Representative images were captured using an LSM800 confocal microscope with a 20 × 0.80 NA objective.

### Transcranial live imaging.

Mice were prepared for cisterna magna tracer infusion, as previously described. The head was shaved, and the dermis was retracted to expose the skull. Throughout the experiment, mice received supplemental oxygen. They were positioned and secured by placing their noses into a nosecone piece on a metal stage. Top-down dorsal skull images were captured every minute using a Leica M165FC stereomicroscope equipped with a 1 × Plan objective, LED fluorescence, Cy5 filter, and DFC7000T camera. Select time point images were converted to 8-bit, uniformly adjusted for intensity, and pseudocolored using the “Fire” lookup tables in Image J. Velocity measurements for CSF tracer at the migrating front were calculated using FrontTracker.m in MATLAB (52). This software computes local velocity at the tracer’s leading edge by measuring the distance it advances between images, following user-defined intensity thresholds, as described in (4). Mean sum intensity was determined by calculating the total mean sum fluorescence of tracer at each time point. After imaging, animals were promptly euthanized via transcardial perfusion to collect brain tissue and skull caps.

### Imaging and quantification of brain CSF perfusion.

After transcardial perfusion with 4% PFA, brains were dissected and postfixed in 4% PFA for 24 hours. Subsequently, they were embedded in 3% agarose (Benchmark Scientific) and sliced into 100 μm sections using a Leica VT-1000 vibratome. To preserve tracer integrity, sections were immediately mounted and imaged using an LSM800 confocal microscope with a 5 × 0.16 NA objective. Z-stacks were captured from 6 sections spanning bregma coordinates +1.2 to –2.5. The percent area fraction of tracer was computed from maximum intensity z-stacks (10 mm step size). Whole-brain sections were uniformly thresholded and analyzed in ImageJ, with threshold values set at 10 for 45 kDa OVA-647 and 28 for 3 kDa dextran. Values were averaged to provide a single data point for each animal.

### Meningeal lymphatic imaging and quantification.

After dorsal or basal skull mount preparation, tissue was imaged using either an LSM700 confocal or an LSM800 confocal with a 10 × 0.45 NA objective. Z-stacks were captured with 10 μm interval step sizes. Raw files were z-projected and processed in ImageJ. Sprouts were manually counted, identified as blunt-ended LYVE1 or Prox1-positive vessels branching off the primary lymphatic tree. Vessel diameters along the TVS and hotspot regions were measured by assessing vessel width in 10 defined areas and averaging the values to obtain 1 representative data point per animal. For P17 time points, the growth distance from the sinus confluence was calculated by measuring the distance from the confluence bottom to the highest point with MLVs present. To determine percent area coverage of vessels and OVA-647 tracer, a single ROI encompassing TVS and the confluence of sinuses was generated. The ROI was placed over the vessels in ImageJ, images were uniformly thresholded, and percent area fraction was measured. The same ROI was used for all adult time points, with a separate ROI for P17 time points.

### Amyloid-β coverage in 5xFAD mice.

Mice were fixed via transcardial perfusion with 4% PFA. Brains were postfixed overnight in 4% PFA at 4°C. Subsequently, brains were embedded in 3% agarose and sectioned into 100 mm–thick slices using a VT1000S vibratome (Leica). Sections were incubated overnight in a primary antibody solution with 5% NGS and 0.3% Triton, followed by an additional overnight incubation in a secondary antibody solution. Maximum intensity z-stacks were acquired with 10 mm step intervals using an LSM800 confocal microscope (Zeiss) equipped with a 10 × 0.45 NA objective. Images were uniformly thresholded, and the percent area fraction of amyloid-β was calculated separately in the hippocampus and dorsal cortex, with a boundary set at the insular cortex. Plaque numbers were determined using the ImageJ “analyze particles” tool, counting puncta of at least 20 mm in size to minimize background noise.

### Yoda1 injections.

Yoda1 (Sigma-Aldrich SML1558) was dissolved in DMSO (0.568 μg/μL) and then diluted to the final working concentration in sterile saline (3:80 dilution). DMSO dissolved 3:80 in saline was considered vehicle for all Yoda1 control experiments. Mice were weighed daily and Yoda1 or vehicle was injected intraperitoneally at 213 μg/kg at 6 PM each day for the duration of experimentation (7 days for P17 mice, 20 days for P30 mice). For 22–24-month-old mice, 1 mg/kg was injected daily for 6 weeks. Prior to perfusion fixation or in vivo tracer injections, animals received an additional dose of Yoda1 or vehicle 1 hour prior to experimentation. Yoda1 and saline vehicle–treated animals were healthy with no changes to body weight. For P30 tracer injections following Yoda1 treatment, 2 *Twist1^+/–^* mice were excluded because suture fusion was absent upon visual inspection following euthanasia.

### Statistics.

Quantifications were performed with investigators masked to genotypes. All statistical analyses were performed using GraphPad Prism software. All studies with 2 comparison groups were analyzed with unpaired 2-tailed *t* test, unless otherwise noted. Experiments with more than 2 groups were analyzed with 1-way ANOVA with Dunnett’s or Tukey’s multiple comparisons test, unless otherwise noted. Box plots show the median (horizontal line), the 25th and 75th percentiles (boxed region), and min/max data points. All data are expressed as mean ± SEM. All *P* values < 0.05 were considered significant.

### Study approval.

Mouse experiments were approved by Rutgers IACUC under protocol PROTO201702623 (MAT).

### Data availability.

Data is available from corresponding author upon request. Values for data points shown in graphs and values behind means are reported in the Supporting Data Values file.

## Author contributions

MJM, PSA, GB, and MAT conceptualized the project. MJM, AJ, AR, JKT, and MAT were responsible for formal analysis. MJM performed data curation, with some assistance by PSA, JW, AJ, and AR. YKH and CCS provided technical assistance and reagents. MAT wrote the original draft of the manuscript. MJM, AR, AJ, and MAT reviewed and edited the manuscript. MAT supervised the project and acquired funding for the project.

## Supplementary Material

Supplemental data

Supporting data values

## Version 1 11/02/2023

In-Press Preview

## Version 2 02/15/2024

Electronic publication
