# Supplementary material for: Piezo1 agonist restores meningeal lymphatic vessels, drainage, and brain-CSF perfusion in craniosynostosis and aged mice
Source: J Clin Invest. 2023 Nov 2;134(4):e171468. doi: 10.1172/JCI171468 (PMC10866656; doi:10.1172/JCI171468)
Supplement: Supplemental data [file jci-134-171468-s060.pdf]

Supporting Material

Supplemental materials and methods

AQP4 Immunofluorescence and quantification: 100μm sections were incubated in Dent's Fix (80% methanol, 20% DMSO) and placed on a rocker for 60 minutes at room temperature to improve antibody penetration. Sections were then co-stained with 1:500 rabbit anti-AQP4 primary antibody and 1:100 GS-lectin 488 conjugate in 5% normal donkey serum (NDS) and 0.3% PBS-triton with 1mM calcium chloride (PBS-T + 1 mM $CaCl_2$). Sections were incubated overnight at 4°C. Following PBS washes, sections were stained with 1:500 donkey anti-rabbit 546 (cat# A10040, ThermoFisher Scientific) secondary antibody in 5% NDS and 0.3% PBS-T + 1 mM $CaCl_2$ for one hour at room temperature. The tissue was washed with PBS and stored in primary antibody solution (without AQP4) at 4°C until imaging to preserve the lectin signal. Sections were imaged with a LSM800 microscope and a 10x 0.45NA objective. Z-stacks were obtained with a 10μm step size. Vessels were randomly selected from the dorsal cortex and uniformly thresholded; each section typically had 8-14 large caliber vessels suitable for analysis. ROIs were selected for single vessels, ensuring at least 95% coverage for lectin. Measurements were obtained by dividing percent area coverage for lectin by the percent area coverage for AQP4. For P17 mice, 10 vessels were selected from a section at the level of the hippocampus. Otherwise, 20 vessels were imaged from two sections, one at the level of the striatum and another at the hippocampus (10/section).

Cell fractionation and western blotting: Blood vessel fractionation from whole brain tissue was performed according to a previous protocol (1). In summary, brains were minced using a scalpel in HBSS with 10mM HEPES. Samples were homogenized by aspirating and pushing out using a 21 G cannula (0.8 mm × 120 mm) on a 2 mL syringe. Homogenates were centrifuged at 2000 x g for 10 minutes at 4°C. The supernatant, containing the parenchyma fraction, was mixed with an equal volume of 2x RIPA buffer and stored at -80°C. The pellet, containing blood vessels and glial endfeet, was resuspended by sonication in 2 mL of buffer 1 with 18% (w/v) dextran (Mr ~ 70,000) and then centrifuged at 4400× g at 4°C for 15 minutes. Brain vessels were collected on a 20μm cell strainer (pluriSelect, Leipzig, Germany) by centrifugation at 200× g for 1 minute in a swinging bucket rotor. Vessels were washed twice by resuspending in 1 mL of buffer 1 with 1% (w/v) BSA (buffer 2) on the strainer, followed by centrifugation. The purified vessels were rinsed in buffer 2 on the strainer, transferred to a 1.5 mL tube, and centrifuged at 2000× g at 4°C for 5 minutes. To

remove BSA, the resulting pellet was resuspended in 1 mL of buffer 1, followed by centrifugation at 2000× g at 4°C for 5 minutes. The supernatant was discarded, and the pellet containing blood vessels was frozen on dry ice and stored at -80°C. The vascular fraction (2mg/sample) was separated on 4-12% Bis-Tris gels (NuPAGE) and transferred to PVDF membranes. Membranes were blocked with 1X TBS, 0.1% Tween-20 with 5% w/v nonfat dry milk and blotted with anti-rabbit AQP4 (Millipore #AB3594). After primary antibody incubation, the blots were stripped (Abcam ab282569) and incubated with mouse β-actin (Sigma #A2228, 1:2000). Following washes, secondary sheep anti-mouse IgG-HRP (Sigma #AC111P) was added in the blocking buffer. Gels were treated with chemiluminescence agent, and blots were exposed using film. Protein quantification was performed with three different biological replicates (whole brains) representing unaffected controls and affected littermates. Quantification was carried out using Image J by placing rectangular boxes on each band and using the Analyze Gels function. The areas under the peaks for AQP4 and the β-actin loading control were selected using the wand tool. The final relative quantity for each sample was calculated by determining the ratio of the sample band to the loading control band.

<u>IBA1 and GFAP staining:</u> *Twist1*$^{+/-}$ and wildtype mice (5-6 months old) underwent transcardial perfusion with 4% PFA. Brains were extracted and postfixed overnight (O/N) at 4°C in 4% PFA. Subsequently, brains were embedded in 3% agarose and sectioned coronally at 100μm. Sections were stained with chicken anti-IBA1 (1:500) and mouse anti-GFAP (1:500) in 5% normal goat serum and 0.3% Triton in PBS, with an overnight incubation at 4°C. After staining, sections were imaged using an LSM800 confocal microscope with 10μm z-steps and a 20x objective (0.8 NA). Images were z-projected, and Iba1-positive cells were counted using FIJI software. The percent area fraction of Iba1 and GFAP1 on whole brain sections was calculated using Fiji software. For morphometric analysis, compressed z-stacks were obtained using a 20x objective (0.8 NA) at 1μm step sizes. Images were z-projected, despeckled, and the unsharp mask filter was applied. Images were uniformly thresholded (min:40, max:255). Three random regions of interest (ROIs) were drawn and cropped. In each ROI, the number of cell bodies was manually counted, and the diameter of each soma was measured using the line tool to find the longest length. The "analyze particles" function (size: 5-infinity) was then applied to each ROI with a mask displayed. The masks were skeletonized and analyzed using "analyze skeleton 2d-3d." Average endpoints per soma, average junctions per soma, and average branch length per soma were calculated by summing the respective values and dividing by the number of soma in each ROI. These values were then averaged to obtain one value per mouse.

References

1. Matthes F, Matuskova, H., Arkelius, K., Ansar, S., Lundgaard, I., Meissner, A. An Improved Method for Physical Separation of Cerebral Vasculature and Parenchyma Enables Detection of Blood-Brain-Barrier Dysfunction. *NeuroSci.* 2021;2(1).

Figure S1

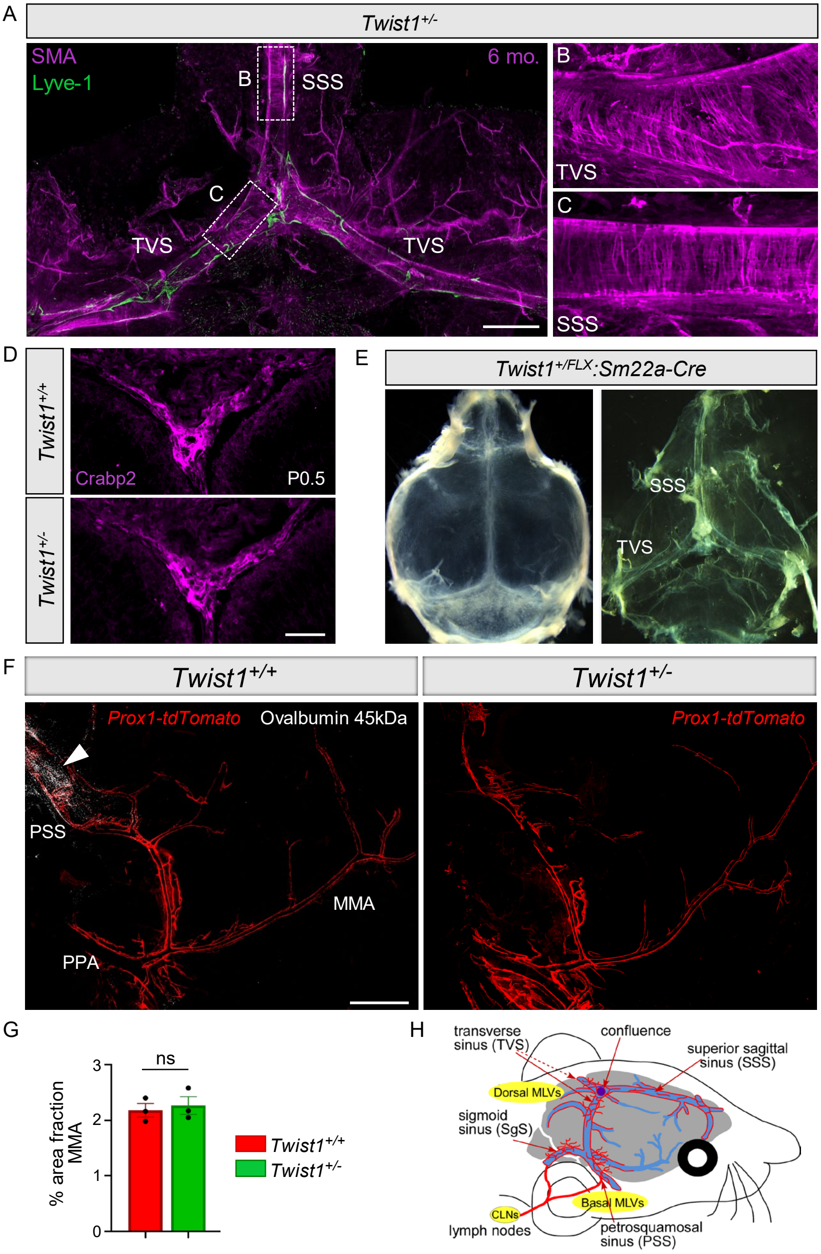

A
Twist1+/-
SMA
Lyve-1
6 mo.
B
SSS
C
TVS
TVS
B
TVS
C
SSS
D
Twist1+/+
Crabp2
P0.5
Twist1+/-
E
Twist1+/FLX:Sm22a-Cre
SSS
TVS
F
Twist1+/+
Twist1+/-
Prox1-tdTomato
Ovalbumin 45kDa
PSS
MMA
PPA
Prox1-tdTomato
G
ns
% area fraction MMA
3
2
1
0
Twist1+/+
Twist1+/-
H
transverse sinus (TVS)
confluence
superior sagittal sinus (SSS)
Dorsal MLVs
sigmoid sinus (SgS)
Basal MLVs
CLNs
lymph nodes
petrosquamosal sinus (PSS)

Figure S1: Dura mater and the venous sinuses are unaffected in *Twist1$^{+/FLX}$:Sm22a-Cre* and *Twist1$^{+/-}$* CS models, and MLVs along the PPA and MMA do not have direct access to CS and develop normally. (A) Dural scraping from a six-month-old *Twist1$^{+/-}$* CS mouse (n=3) with regressed dorsal MLVs. The transverse sinuses are both present. Smooth muscle coverage is normal on the transverse (B, TVS) and superior sagittal sinuses (C, SSS). (D) Coronal cross section at the dorsal midline of a P0.5 mouse labeled with Crabp2, which marks dura and the underlying arachnoid. The dura and arachnoid membranes show normal Crabp2 expression and are not hypoplastic in *Twist1$^{+/-}$* CS mice. (E) Dorsal craniotomy and dural flat mount. The dura is easily peeled off the skull in *Twist$^{+/-}$* and *Twist1$^{+/FLX}$:Sm22a-Cre* mice (shown). Representative images from young adult mice aged 2-4 months. (F and G) The percent area fraction of *Prox1-tdTomato* signal along the MMA is normal in *Twist1$^{+/-}$* CS mice compared with unaffected littermates [*Twist1$^{+/+}$* (n=3); *Twist1$^{+/-}$* (n=3)]. Note that tracer is present (arrowhead) in perisinusoidal dura enveloping the PSS (petrosquamosal sinus) but is absent in dura surrounding the PPA (pterygopalatine artery) and MMA (middle meningeal artery). (H) Schematic overview of dorsal and basal perisinusoidal MLVs in mouse. ns, non-significant, *two-tailed unpaired t test.* Scale bar: (A) 250μm; (D) 50μm; (F) 1mm.

Figure S2

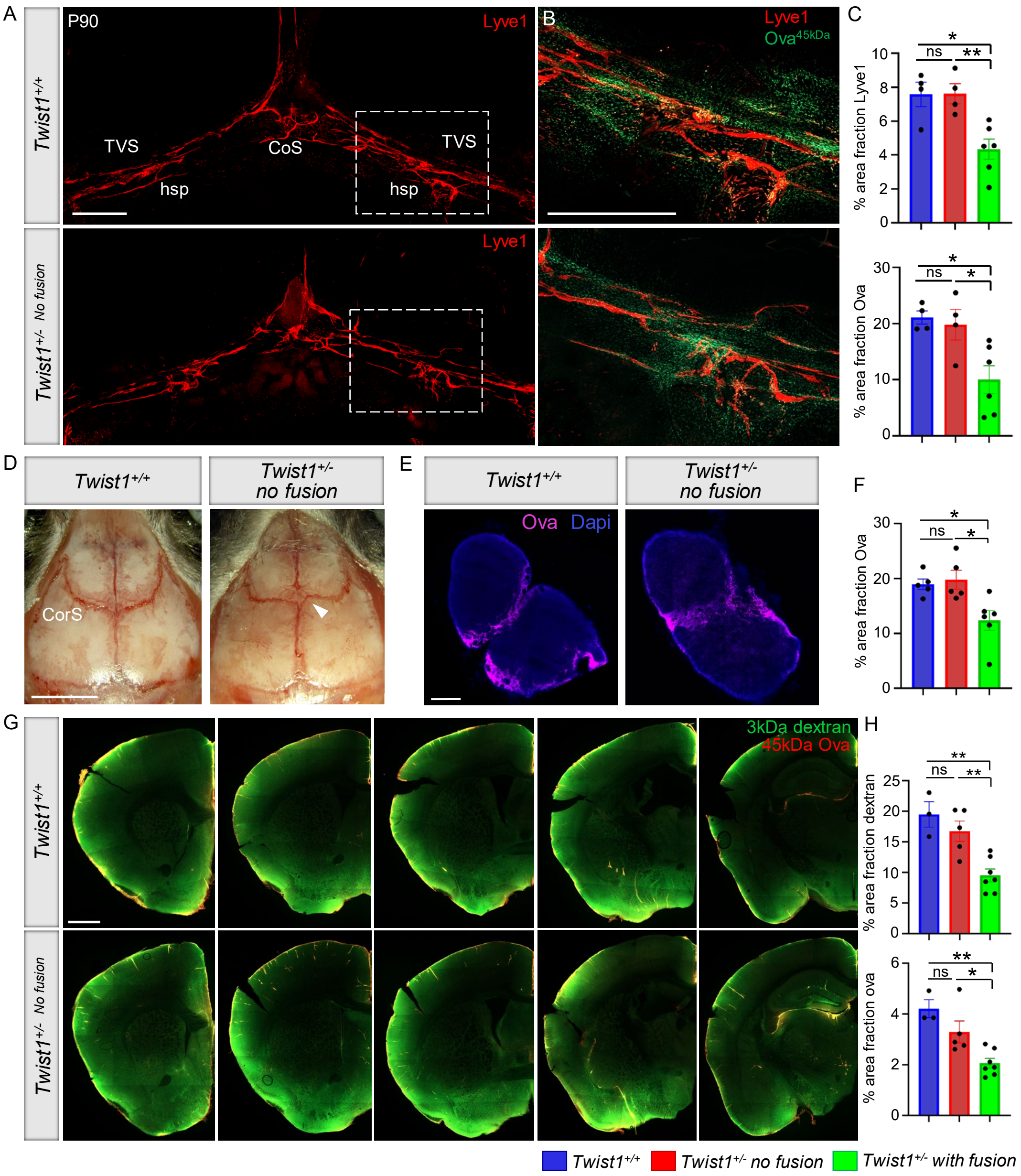

A
Twist1+/+
P90
Lyve1
TVS
CoS
TVS
hsp
hsp
B
Lyve1
Ova45kDa
C
% area fraction Lyve1
ns
**
*
Twist1+/- No fusion
Lyve1
% area fraction Ova
D
Twist1+/+
Twist1+/- no fusion
CorS
E
Twist1+/+
Twist1+/- no fusion
Ova Dapi
F
% area fraction Ova
G
Twist1+/+
Twist1+/- No fusion
3kDa dextran
45kDa Ova
H
% area fraction dextran
% area fraction ova
Twist1+/+
Twist1+/- no fusion
Twist1+/- with fusion

Figure S2: MLV networks, flow to perisinusoidal dura, drainage to the dCLNs, and brain-CSF perfusion are unaffected in young adult *Twist1$^{+/-}$ mice* without suture fusion. (A) Representative images show that dorsal MLV networks, stained with Lyve-1, and hotspots along the transverse sinus (TVS) develop normally in *Twist1$^{+/-}$* mice that lack coronal suture fusion. (B) Following injection of 45kDa ovalbumin tracer into the cisterna magna, tracer deposition in perisinusoidal dura surrounding MLVs and hotspots is comparable between controls and *Twist1$^{+/-}$* mice that lack fusion of the coronal sutures, versus *Twist1$^{+/-}$* mice that have coronal suture synostosis. (C) Quantification of percent area fraction Lyve-1 and ovalbumin 45kDa [*Twist1$^{+/+}$* (n=4); *Twist1$^{+/-}$* no fusion (n=5); *Twist1$^{+/-}$* fusion (n=6)]. (D) Skull overviews showing patent coronal sutures (CorS) in a *Twist1$^{+/-}$* mouse. The arrowhead denotes that the orientation of the coronal sutures is more 'box-like' compared to wildtype controls. (E) Following injection of 45kDa ovalbumin tracer into the cisterna magna, drainage to the dCLNs is normal in *Twist1$^{+/-}$* mice that lack coronal suture fusion, whereas *Twist1$^{+/-}$* mice with coronal suture synostosis have a significant reduction. (F) Quantification of percent area fraction of 45kDa tracer [*Twist1$^{+/+}$* (n=5); *Twist1$^{+/-}$* no fusion (n=5); *Twist1$^{+/-}$* fusion (n=6)]. (G) Following injection of 3kDa Dextran and 45kDa ovalbumin tracer into the cisterna magna, tracer perfusion into the brain is comparable between wildtype controls and *Twist1$^{+/-}$* mice that lack coronal suture fusion or have very mild partial unilateral fusion, versus *Twist1$^{+/-}$* mice with near-complete unilateral or bilateral fusion of the coronal sutures. (H) Quantification of percent area fraction of 3kDa Dextran and 45kDa ovalbumin tracer in brain tissue [*Twist1$^{+/+}$* (n=3); *Twist1$^{+/-}$* no fusion (n=5); *Twist1$^{+/-}$* fusion (n=7)]. ns, non-significant, **$p \leq 0.05$, **$p \leq 0.01$ One way ANOVA with Tukey's multiple comparison test*. Scale bar: (A and B) 1mm; (D) 5mm; (E) 200μm; (F) 1mm

Figure S3

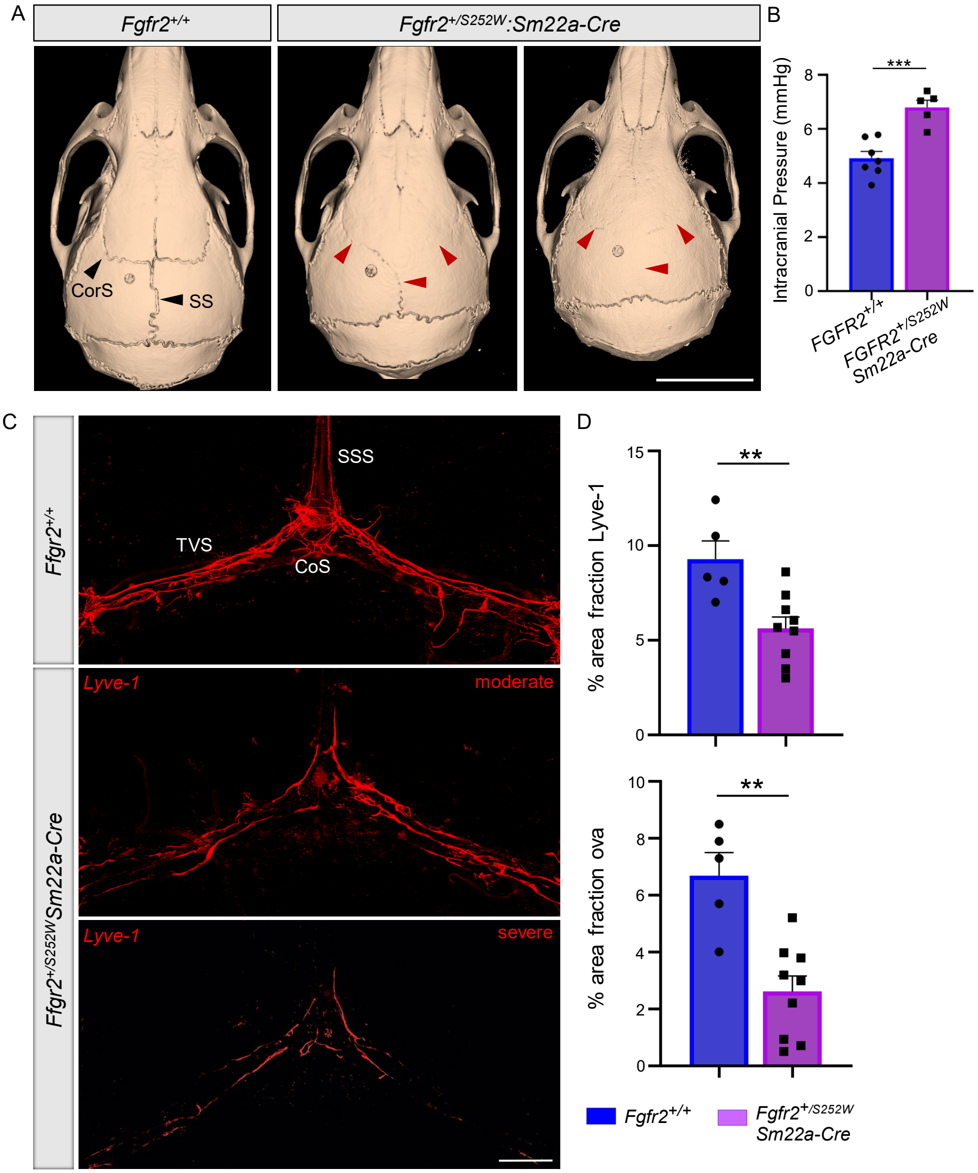
A
Fgfr2+/+
Fgfr2+/S252W:Sm22a-Cre
CorS
SS
B
Intracranial Pressure (mmHg)
***
FGFR2+/+
FGFR2+/S252W Sm22a-Cre
C
Ffgr2+/+
SSS
TVS
CoS
Lyve-1
moderate
Lyve-1
severe
Ffgr2+/S252WSm22a-Cre
D
% area fraction Lyve-1
**
% area fraction ova
**
Fgfr2+/+
Fgfr2+/S252W Sm22a-Cre

Figure S3: *Fgfr2$^{+/S252W}$:Sm22a-Cre* mice have CS, raised intracranial pressure, and hypoplastic dorsal MLVs. (A) Representative reconstructed computed tomography (CT) scans from two-month-old adults showing normal skull and suture morphology in *Fgfr2$^{+/+}$* control versus *Fgfr2$^{+/S252W}$:Sm22a-Cre* mice with CS. (Middle image) Near-complete and complete fusion of the left and right coronal sutures (arrowheads), respectively, with partial fusion of the sagittal suture (arrowhead). (Right image) Complete fusion of the sagittal suture and bilateral fusion of the coronal sutures. (A') Intracranial pressure is significantly elevated in *Fgfr2$^{+/S252W}$:Sm22a-Cre* mice with CS [*Fgfr2$^{+/+}$* (n=7); *Fgfr2$^{+/S252W}$:Sm22a-Cre* (n=5)]. (B) Representative images showing hypoplasia of dorsal MLVs in *Fgfr2$^{+/S252W}$:Sm22a-Cre* mice with CS. (B') Quantification of percent area fraction Lyve-1 and ovalbumin 45kDa [*Fgfr2$^{+/+}$* (n=5); *Fgfr2$^{+/S252W}$:Sm22a-Cre* (n=9)]. CorS=coronal suture; SS=sagittal suture; TVS=transverse sinus; CoS=confluence of sinuses; SSS=superior sagittal sinus. ****$p \leq 0.001$. two-tailed unpaired t test with Welch's correction.* Scale bar: (A) 5mm; (C) 1mm.

Figure S4

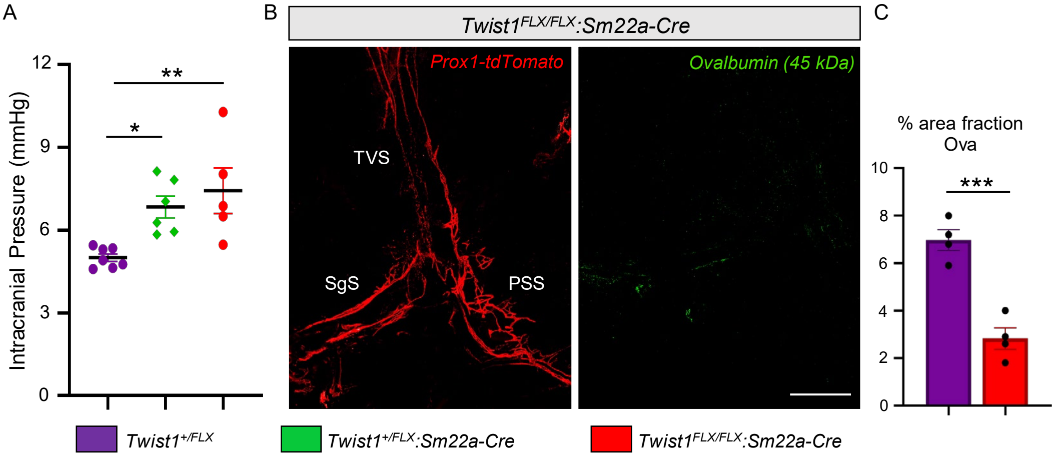
A
B
C
Intracranial Pressure (mmHg)
12
9
6
3
0
**
*
Twist1FLX/FLX:Sm22a-Cre
Prox1-tdTomato
Ovalbumin (45 kDa)
TVS
SgS
PSS
% area fraction
Ova
10
8
6
4
2
0
***
Twist1+/FLX
Twist1+/FLX:Sm22a-Cre
Twist1FLX/FLX:Sm22a-Cre

Figure S4: Homozygous *Twist1$^{+/FLX}$:Sm22a-Cre* mice have raised intracranial pressure and reduced CSF flow to perisinusoidal dura. (A) Similar to heterozygous *Twist1$^{+/FLX}$:Sm22a-Cre* mice, homozygous *Twist1$^{FLX/FLX}$:Sm22a-Cre* mice with CS have raised intracranial pressure [*Twist1$^{FLX/+}$* (n=7); *Twist1$^{FLX/+}$:Sm22a-Cre* (n=6); *Twist1$^{FLX/FLX}$:Sm22a-Cre* (n=5)]. (B) Representative image showing lack of ovalbumin 45kDa tracer in perisinusoidal dura surrounding MLVs in a 2-month-old *Twist1$^{FLX/FLX}$:Sm22a-Cre* mouse. (C) Quantification of percent area fraction of ovalbumin 45kDa tracer in perisinusoidal dura [*Twist1$^{FLX/+}$* and *Twist1$^{FLX/FLX}$:Sm22a-Cre* (n=4)]. TVS=transverse sinus; SgS=sigmoid sinus; PSS=petrosquamosal sinus. *$p \leq 0.05$, **$p \leq 0.01$, ***$p \leq 0.001$ *One way ANOVA with Tukey's multiple comparison test* (A) and *two-tailed unpaired t test* (B). Data points in A reflecting *Twist1$^{FLX/+}$* and *Twist1$^{FLX/+}$:Sm22a-Cre* mice are from Figure 1. Scale bar=1mm

Figure S5

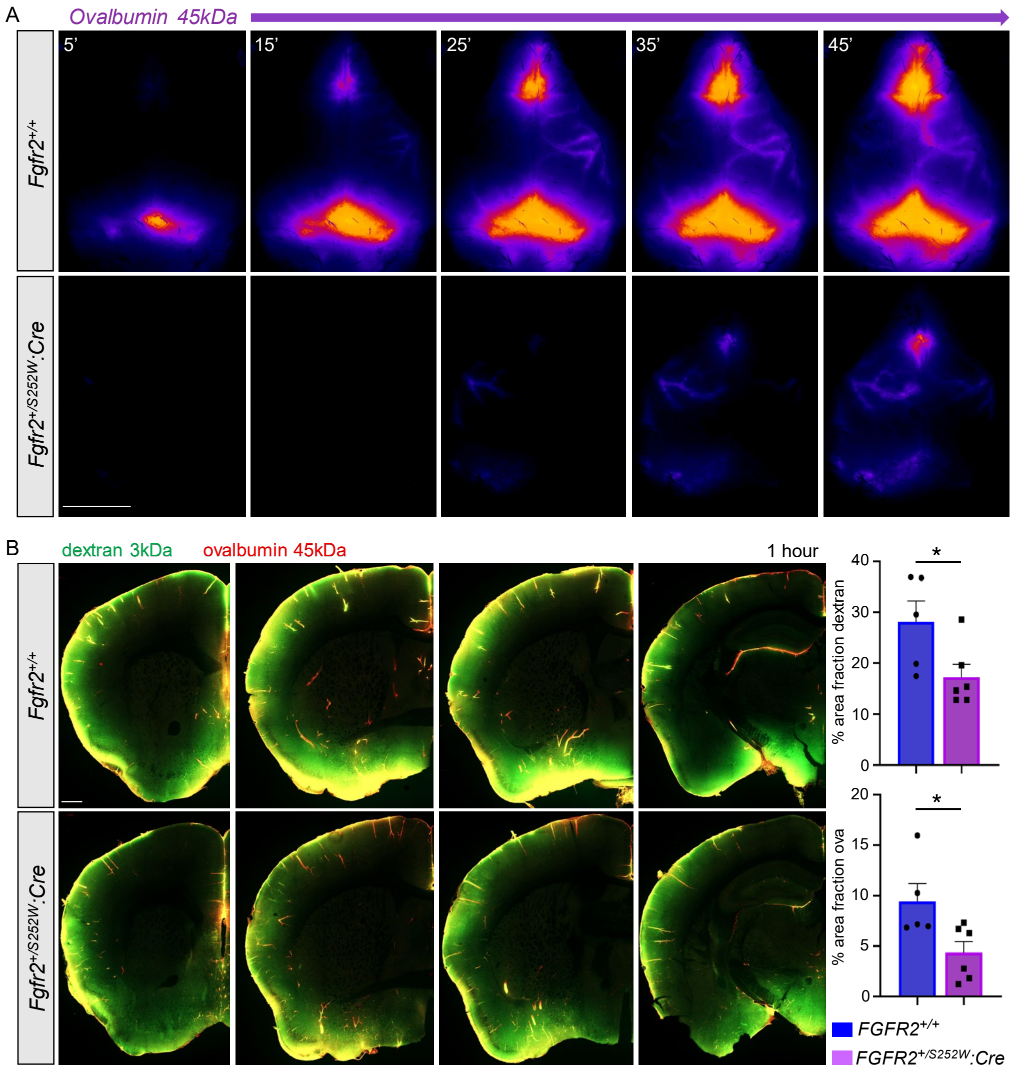
A
Ovalbumin 45kDa
5'
15'
25'
35'
45'
Fgfr2+/+
Fgfr2+/S252W:Cre
B
dextran 3kDa
ovalbumin 45kDa
1 hour
Fgfr2+/+
Fgfr2+/S252W:Cre
% area fraction dextran
% area fraction ova
*
0
10
20
30
40
0
5
10
15
20
FGFR2+/+
FGFR2+/S252W:Cre

Figure S5: *Fgfr2$^{+/S252W}$:Sm22a-Cre* mice with CS have altered CSF flow with reduced perfusion of CSF macromolecules into the brain. (A) Representative images taken during transcranial live imaging of a 45kDa ovalbumin tracer injected into the cisterna magna of young adult mice. Compared to unaffected littermates (top panel), *Fgfr2$^{+/S252W}$:Sm22a-Cre* mice (bottom panel) show perturbations to CSF tracer flow along preferred dorsal pathways, similar to what is observed in *Twist1$^{+/-}$* mice. (B) Representative images show that perfusion of CSF macromolecules into the brain is reduced in *Fgfr2$^{+/S252W}$:Sm22a-Cre* mice. (Right) Quantification of percent area fraction of 3kDa Dextran and 45kDa ovalbumin tracer in brain tissue [*Fgfr2$^{+/+}$* (n=5); *Fgfr2$^{+/S252W}$:Sm22a-Cre* (n=6)]. *$p \leq 0.01$ *two-tailed unpaired t test*. Scale bar: (A) 5mm; (B) 500μm

Figure S6

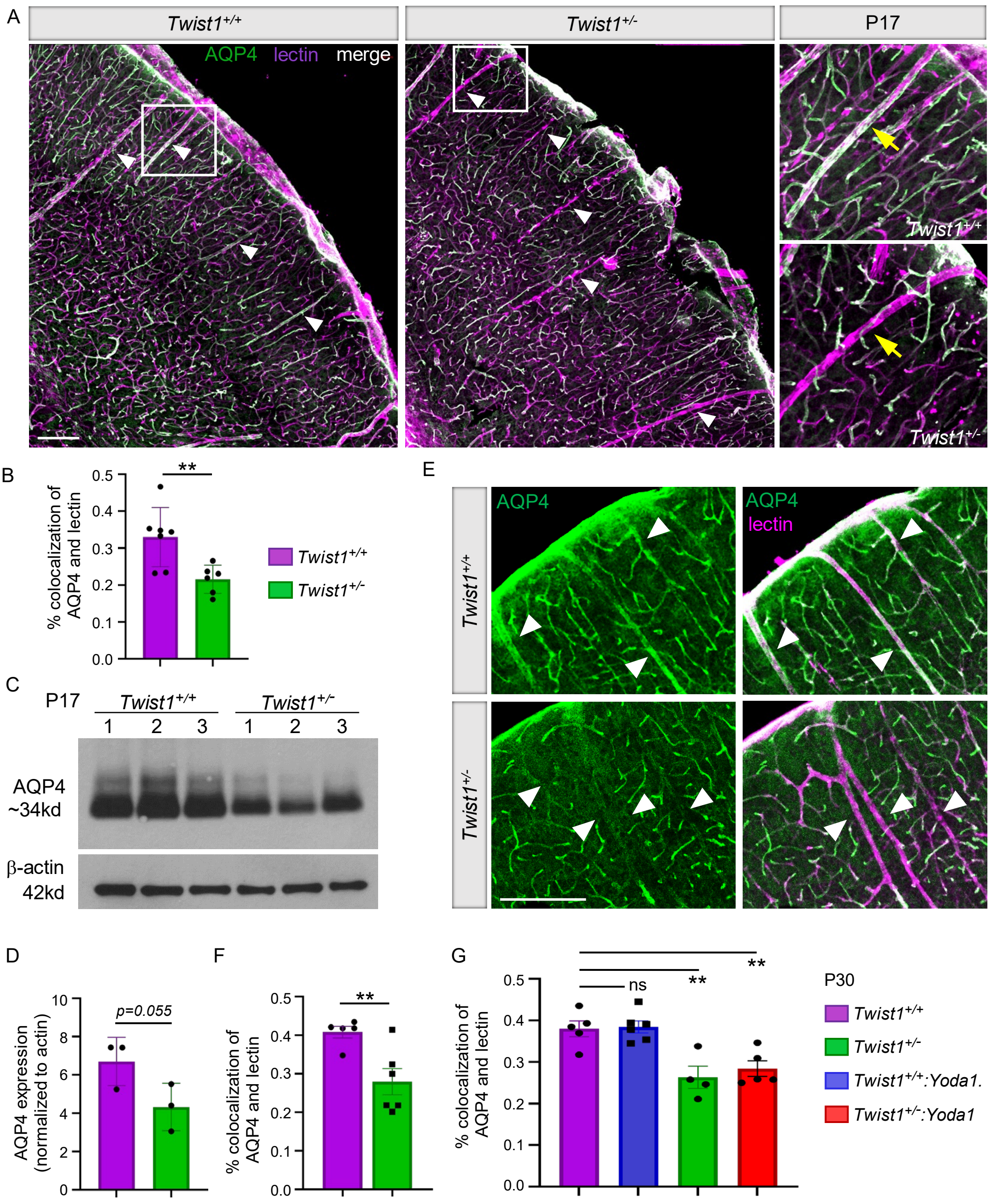
A
Twist1+/+
Twist1+/-
P17
AQP4 lectin merge
Twist1+/+
Twist1+/-
B
% colocalization of AQP4 and lectin
0.5
0.4
0.3
0.2
0.1
0.0
**
Twist1+/+
Twist1+/-
C
P17
Twist1+/+
Twist1+/-
1 2 3 1 2 3
AQP4 ~34kd
β-actin 42kd
E
Twist1+/+
Twist1+/-
AQP4
AQP4 lectin
D
AQP4 expression (normalized to actin)
10
8
6
4
2
0
p=0.055
F
% colocalization of AQP4 and lectin
0.5
0.4
0.3
0.2
0.1
0.0
**
G
% colocalization of AQP4 and lectin
0.5
0.4
0.3
0.2
0.1
0.0
ns
**
**
P30
Twist1+/+
Twist1+/-
Twist1+/+:Yoda1.
Twist1+/-:Yoda1

Figure S6: AQP4 is reduced at glial endfeet that wrap around large-caliber vessels. (A) Coronal sections through the dorsolateral cortex in P17 juveniles. The coverage of AQP4 protein along large-caliber blood vessels labeled with GS-lectin IB4 is reduced in *Twist1*$^{+/-}$ mice (arrowheads). (B) Quantification of percent colocalization of AQP4 protein and lectin [*Twist1*$^{+/+}$ (n=7); *Twist1*$^{+/-}$ (n=6)]. (C) Protein fractions from P17 juvenile mice containing glial endfeet tethered to blood vessels show a reduction in AQP4 protein. (D) Quantification of AQP4 in the vascular fraction (normalized to actin) from three *Twist*$^{+/+}$ and three *Twist1*$^{+/-}$ brains [*Twist1*$^{+/+}$ and *Twist1*$^{+/-}$ (n=3)]. (E) In young *Twist1*$^{+/-}$ adult mice with CS, AQP4 coverage is still reduced along large-caliber vessels (arrowheads). (F) Quantification of percent colocalization of AQP4 protein and lectin [*Twist1*$^{+/+}$ (n=5); *Twist1*$^{+/-}$ (n=6)]. (G) Quantification of percent colocalization of AQP4 protein and lectin in P30 mice treated with saline vehicles or Yoda1. *Twist1*$^{+/-}$*:Yoda1* mice still show a significant reduction of AQP4 protein along large-caliber blood vessels labeled with GS-lectin IB4 compared with controls receiving Yoda1 or saline vehicle [*Twist1*$^{+/+}$ vehicle (n=5); *Twist1*$^{+/+}$ Yoda1 (n=6); *Twist1*$^{+/-}$ vehicle (n=4); *Twist1*$^{+/-}$ Yoda1 (n=5)]. (A', B', C') **$p \leq 0.01$ *two-tailed unpaired t test*. (D) **$p \leq 0.01$ *one-way ANOVA with Dunnett's multiple comparison test*. Scale bar: (A and E) 200μm

Figure S7

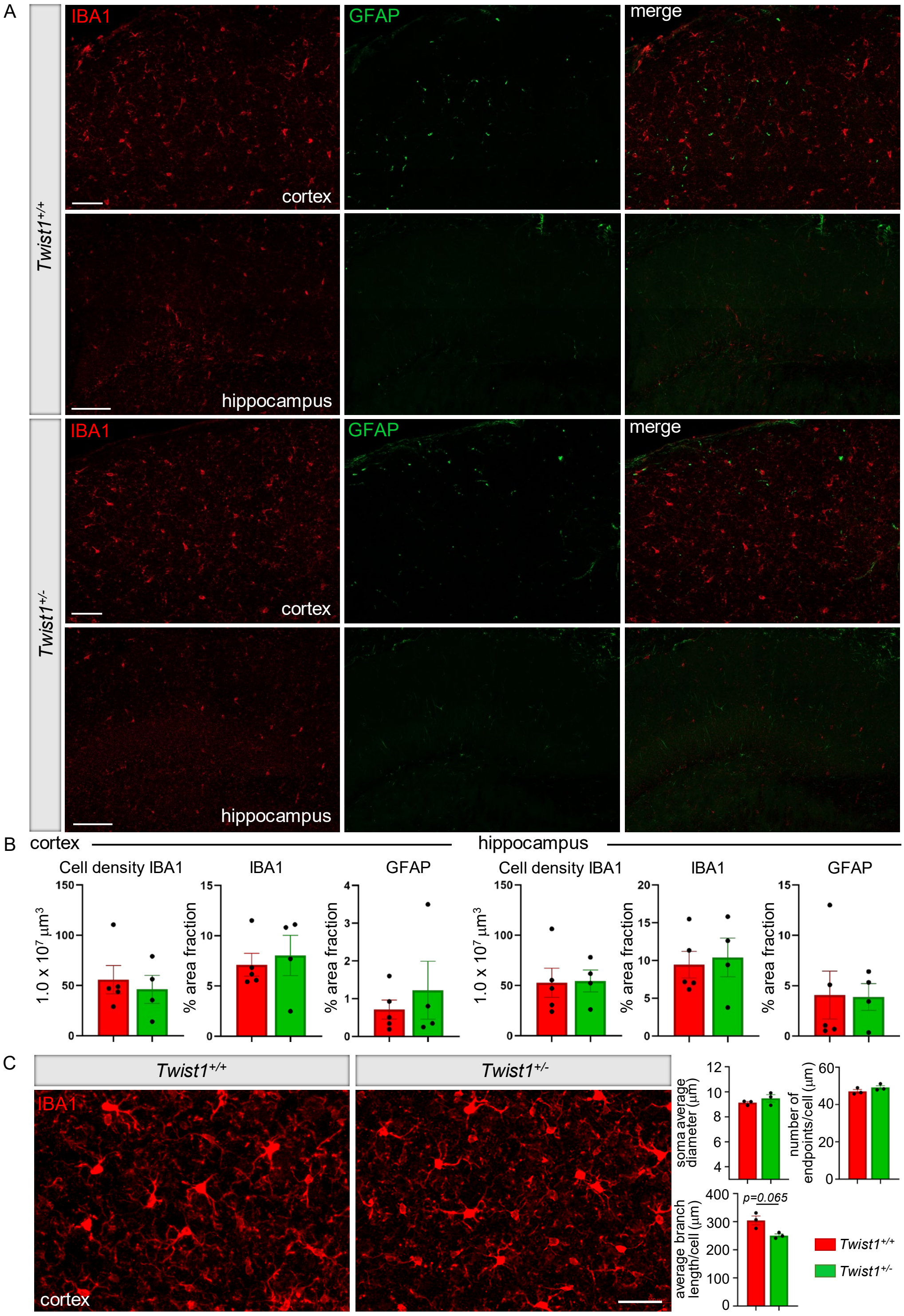

A
IBA1
GFAP
merge
cortex
hippocampus
Twist1+/+
Twist1+/-
B
cortex
hippocampus
Cell density IBA1
IBA1
GFAP
1.0 x 10⁷ µm³
% area fraction
C
soma average diameter (µm)
number of endpoints/cell (µm)
average branch length/cell (µm)
p=0.065
cortex
Twist1+/+
Twist1+/-

Figure S7: *Twist1$^{+/-}$* CS mice do not show changes to astro- or microgliosis. (A) Representative coronal sections through the cortex and hippocampus stained with GFAP and IBA1. (B) No changes to astro- or microgliosis are detected in *Twist1$^{+/-}$* craniosynostosis mice, as measured by the percent area fraction of GFAP and IBA1 staining compared to control littermates [*Twist1$^{+/+}$* (n=5); *Twist1$^{+/-}$* (n=4)]. (C) Representative images of microglia in the cortex at higher magnification. No significant changes to morphology are detected [*Twist1$^{+/+}$* (n=3); *Twist1$^{+/-}$* (n=3)]. *two-tailed unpaired t test with Welch's correction*. Scale bar: (A) 50μm; (C) 100μm
